# Mesoscale Simulations Reveal How Salt Influences Clay Particles Agglomeration in Aqueous Dispersions

**DOI:** 10.1021/acs.jctc.3c00719

**Published:** 2023-11-02

**Authors:** Tran Thi Bao Le, Aaron R. Finney, Andrea Zen, Tai Bui, Weparn J. Tay, Kuhan Chellappah, Matteo Salvalaglio, Angelos Michaelides, Alberto Striolo

**Affiliations:** † Department of Chemical Engineering, University College London, WC1E 7JE, London, United Kingdom; ‡ Dipartimento di Fisica Ettore Pancini, Università di Napoli Federico II, Monte S. Angelo, I-80126 Napoli, Italy; § BP Exploration Operating Co. Ltd, Chertsey Road, Sunbury-on-Thames TW16 7LN, United Kingdom; ∥ Yusuf Hamied Department of Chemistry, University of Cambridge, Lensfield Road, Cambridge CB2 1EW, United Kingdom; ⊥ School of Sustainable Chemical, Biological and Materials Engineering, The University of Oklahoma, Norman, Oklahoma 73019, United States

## Abstract

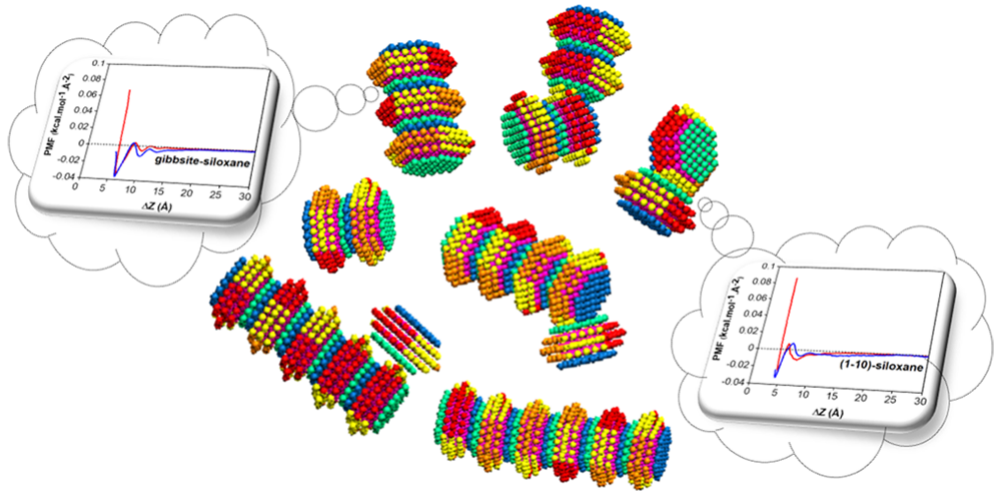

The aggregation of clay particles is an everyday phenomenon of scientific and industrial relevance. However, it is a complex multiscale process that depends delicately on the nature of the particle–particle and particle–solvent interactions. Toward understanding how to control such phenomena, a multiscale computational approach is developed, building from molecular simulations conducted at atomic resolution to calculate the potential of mean force (PMF) profiles in both pure and saline water environments. We document how it is possible to use such a model to develop a fundamental understanding concerning the mechanism of particle aggregation. For example, using molecular dynamics simulations conducted at the mesoscale in implicit solvents, it is possible to quantify the size and shape of clay aggregates as a function of system conditions. The approach is used to emphasize the role of salt concentration, which directly affects the potentials of the mean forces between kaolinite particles. While particle agglomeration in pure water yields large aggregates, the presence of sodium chloride in the aqueous brine leads instead to a large number of small aggregates. These results are consistent with macroscopic experimental observations, suggesting that the simulation protocol developed could be relevant for preventing pore blocking in heterogeneous porous matrixes.

## 1 Introduction

Because the aggregation of particles and nanoparticles is important for a variety of practical applications, it has attracted much fundamental research attention over many years. For example, it has been shown that, under appropriate conditions, nanocrystals can self-assemble into free-floating sheets,^1^ nanoparticles can yield spherulites and short rods,^2^ and isotropic particles can form anisotropic structures because of a balance between enthalpic and entropic effects.^3^ The assembly of particles at interfaces has been shown to affect surface tension and rheological properties,^4,5^ and nanoparticles can be designed to fuse with lipid bilayers.^6^ Particles agglomeration can also occur within porous materials,^7,8^ where it might be possible to direct particles assembly by tuning the flow field, and where particle deposition on the pore surface can control the particles residence time within the porous network.^9^ When particles agglomerate uncontrollably in a porous material, they can affect the transport of fluids through said material, with potential negative effects in applications such as catalysis and carbon sequestration. For example, clay minerals (e.g., smectite, kaolinite, Illite, and chlorite) are thought to be responsible for formation damage in sandstone reservoirs.^10^ Indeed, clay particles can agglomerate, deposit, and block pore throats, ultimately affecting hydrocarbon production as well as access to the pore space in geological carbon sequestration projects.^11,12^

The prediction of particle agglomeration into self-assembled structures has been addressed by several coarse-graining approaches. Indeed, coarse-grained (CG) molecular dynamics is emerging as a powerful tool to study large-sized and complex systems for time scales of up to milliseconds. Compared with all-atom models, CG representations reduce the number of degrees of freedom by mapping small groups of atoms into simple CG particles. As a result, coarse-grained models require less computational power and allow for much faster sampling than their atomistic counterparts. The field is evolving quickly. For example, Ledum et al.^13^ proposed a hybrid particle-field molecular dynamics approach that allows for on-the-fly increase of the CG length, reactive CG simulations have been developed based on the MARTINI force field,^14^ the CG simulation in implicit solvents has been conducted for several systems, including block copolymers,^15^ and protocols have been proposed for coarse-graining the effective interactions between cubic nanoparticles due to functionalization with DNA fragments,^16^ as well as for a potential-matching method across the length scales, demonstrated for systems containing dipalmitoylphosphatidylcholine (DPPC) lipids.^17^ The results from these approaches reveal important fundamental insights. For example, the CG simulations of DNA-functionalized particles showed nonadditive mixing of colloidal diamond phase formation,^18^ and a predictive model has been developed for pH-dependent reversible CG self-assembly of bioinspired nanoparticles.^19^ A protocol for conducting CG molecular simulations is developed here and applied to describe the anisotropic agglomeration of kaolinite platelets in aqueous systems.

Kaolinite particles, abundant in untreated sandstone, often occur as discrete aggregates. In a previous study,^20^ we quantified the effective potential of mean force (PMF) between two kaolinite particles in an aqueous solution as a function of salt content using atomistic simulations. Consistent with other findings in the literature,^21^ our results suggest that the interactions between the nanoparticles vary from attractive to repulsive, depending on the relative orientation among the nanoparticles, the distance between the interacting nanoparticles, and on the salt content in the aqueous brine. In the saline solution, ions demonstrated a tendency to preferentially adsorb onto the basal facets of the particles. This countered the inherent dipole present across the kaolinite particles. Consequently, the ions screen the electrostatic interparticle interaction, leading to noticeably diminished long-range PMF profiles compared to those observed in pure water. Our prior study aimed at building the foundation for further investigating the aggregation of clay particles via numerical simulations. Although achieving a description of clay particle agglomeration at the atomic scale is desirable, such an ambition is challenged by the available computing resources. Hence, a CG approach is chosen for the present contribution. The challenge is to retain within the CG model the atomistic features that are thought to be responsible for the macroscopic observables, in our case, the agglomeration mechanism. The idea of coarse-graining clay minerals has been discussed in several studies,^22−28^ and different algorithms have been attempted. For example, Zu et al.^22^ conducted mesoscale simulations using models based on PMF interactions to investigate the aggregation of imogolite nanotubes by mapping them onto a series of spherical subparticles. Ebrahimi et al.^23^ examined the mesoscale aggregation and mechanical properties of Wyoming Na-montmorillonite platelets. The mesoscale simulations were performed by using the Gay–Berne potential calibrated from full atomistic simulations. Similarly, Bandera et al.^26^ studied the compressive behavior of kaolinite platelets treated as flat (3D) ellipsoidal particles whose interactions were described by a modified form of the Gay–Berne potential, calibrated against the Derjaguin–Landau–Verwey–Overbeek (DLVO)^29^ theory. The present work stems from the hypothesis that the most relevant atomistic signatures are encoded into the anisotropic PMF profiles associated with facet-dependent interactions of kaolinite particles and are attainable via atomistic simulations.^20^ We conduct CG simulations to test whether the aggregates produced according to such a hypothesis are representative of those observed experimentally.

We developed a computationally efficient CG model of kaolinite primary particles, described as rigid coarse-grained hexagonal nanostructures, to test our hypothesis. The innovations introduced in the proposed approach, compared to prior contributions, include the fact that anisotropic PMF profiles from atomistic simulations were matched with the fitted CG force fields. This allows our approach to describe differences between gibbsite–siloxane, siloxane–siloxane, and gibbsite–gibbsite potentials, for example, which are not immediate when CG interactions are fitted with potentials such as Gay–Berne. To properly describe anisotropic interactions, which in our hypothesis is important to predict the aggregation of clay platelets, we devised an approach to maintain the three-dimensionality of a single platelet, which required having structural CG beads at the interior of the platelets to maintain rigidity and describing CG beads on facets and edges of the platelets with different potentials. To ensure that the atomistic PMF profiles are reproduced by the CG potentials, the free energy is normalized by the density of CG beads on an interface, which allows our model to describe the assembly of platelets with a variety of size distributions.

The PMF profiles obtained via atomistic MD simulations were fitted to analytical models; the latter were then used to conduct implicit-solvent CG simulations within the Langevin formalism. Consistent with prior studies, it is demonstrated herein that this approach allows us to achieve moderately long time scales with reasonable computing time resources. Building on recent developments, particle aggregation was quantified under various conditions by using a graph-based clustering protocol. The results were qualitatively compared against macroscopic observations, suggesting that the mesoscale approach could be useful for identifying conditions conducive to aggregation and precipitation. In addition, analysis of the size of the agglomerates as a function of time allows us to discuss the relevance of our model with respect to the nucleation and growth of large aggregates and, in particular, to appreciate the effect of salt on the macroscopic observables. Although the innovations introduced could be considered as incremental from the point of view of algorithm development, the quantities that can be extracted from the analysis of the simulated CG Langevin dynamics trajectories allow us to extract information about nucleation, growth, and dissociation of platelet aggregates, which is of fundamental importance for the investigation of self- and controlled assembly in dispersions.

The remainder of the manuscript is organized as follows: we first describe the simulation method; we then present some of the most significant results obtained with the coarse-grained simulations; and we finally summarize our findings, illustrating some suggestions for future research targeted to relate the likelihood of particles aggregation into anisotropic self-assembled structures, with a variety of applications ranging from materials science to biology, from the prevention of pore blocking in geological formations as well as in catalysts.

## 2 SIMULATION METHODS

### 2.1 Coarse-Grained Model

**Figure 1:**
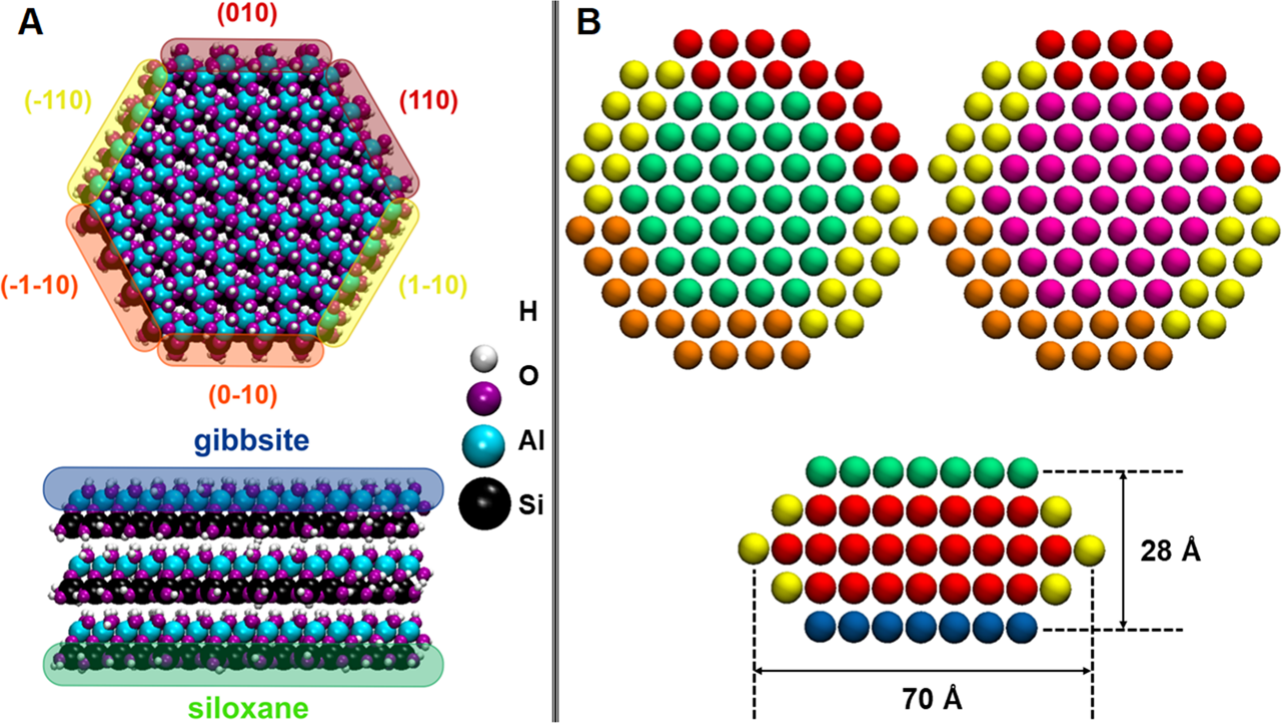
Panel A: Atomistic model of the kaolinite platelet simulated in our prior work indicated gibbsite and siloxane surfaces as well as crystallographic terminations. White, purple, cyan, and black spheres represent the hydrogen, oxygen, aluminum, and silicon atomic species, respectively. Panel B: Schematic coarse-grained representation of the CG kaolinite nanostructure simulated here, showing plane and side views. Gibbsite and siloxane surfaces are shown as blue and green spheres, respectively. Red, yellow, and orange spheres represent (010), (1–10), and (0–10) edges, respectively. Dummy spheres are colored pink; they can be seen in the interior of the kaolinite nanostructures.

The structure of kaolinite particles is often observed as an approximately hexagonal platelet.^30^ Hence, in this study, kaolinite particles were coarse-grained into a collection of beads rigidly assembled, yielding flat, hexagonal nanostructures. Because our prior atomistic simulations revealed that kaolinite–kaolinite interactions in aqueous systems are highly anisotropic, it is necessary to develop a CG model to capture such anisotropic interactions. In particular, three different face-to-face relative orientations (gibbsite–gibbsite, siloxane–siloxane, and gibbsite–siloxane) need to be considered. Our prior work also identified six possible edge–face relative orientations, although those interactions were found to be much weaker compared to the face-to-face ones. To reflect this level of anisotropy, five types of spheres were used to build the CG kaolinite nanostructures, differentiating two nonequivalent basal faces and six edges. These differences are highlighted using different colors in Figure 1. Type 1 and Type 2 spheres are hexagonally arranged to form monolayers representing gibbsite (G) and siloxane (S) faces of kaolinite, respectively. Type 3, Type 4, and Type 5 spheres are used to model the (010), (1–10), and (0–10) edges, respectively. The latter three types of spheres are sandwiched between gibbsite and siloxane layers in a terraced fashion. The resultant model is similar to the one developed by de Bono and McDowell,^31^ except that these authors constructed a kaolinite model from one layer of spheres, effectively building a flat platelet, with additional smaller spheres placed in a staggered “zigzag” manner along the platelet edge.

Instead of considering a flat platelet, we used dummy spheres to fill the inner space of the nanostructures bordered by face and edge spheres. This was required to avoid the computational inefficiencies observed during our CG simulations. The interactions between the dummy spheres and other spheres are excluded from the integration of the equations of motion. It should be noticed that six spheres at the corners of the middle layer are removed such that the number of edge spheres (i.e., Type 3, 4, and 5 spheres) that contribute to edge–face interactions remains the same in all directions.

The center-to-center distance between spheres within a kaolinite nanostructure is 7 Å. Hence, the layered arrangement described in Figure 1 results in a hexagonal nanostructure with a thickness of 28 Å, which can rotate and translate as a rigid body. Each nanostructure comprises 282 spheres yielding a maximum diameter (corner-to-corner distance) of 70 Å.

The approach of modeling clay platelets with a rigid array of spheres has been used in several prior studies.^24,31−33^ However, the platelets in those prior studies were constructed from one or two layers of one or two types of subparticles. A terrace structure, along with different subparticle types, can allow for anisotropic interactions between the various surfaces to be implemented, potentially expanding the range of supra-molecular structures that can be observed during the simulations.

### 2.2 Development of the CG Force Field

**Figure 2:**
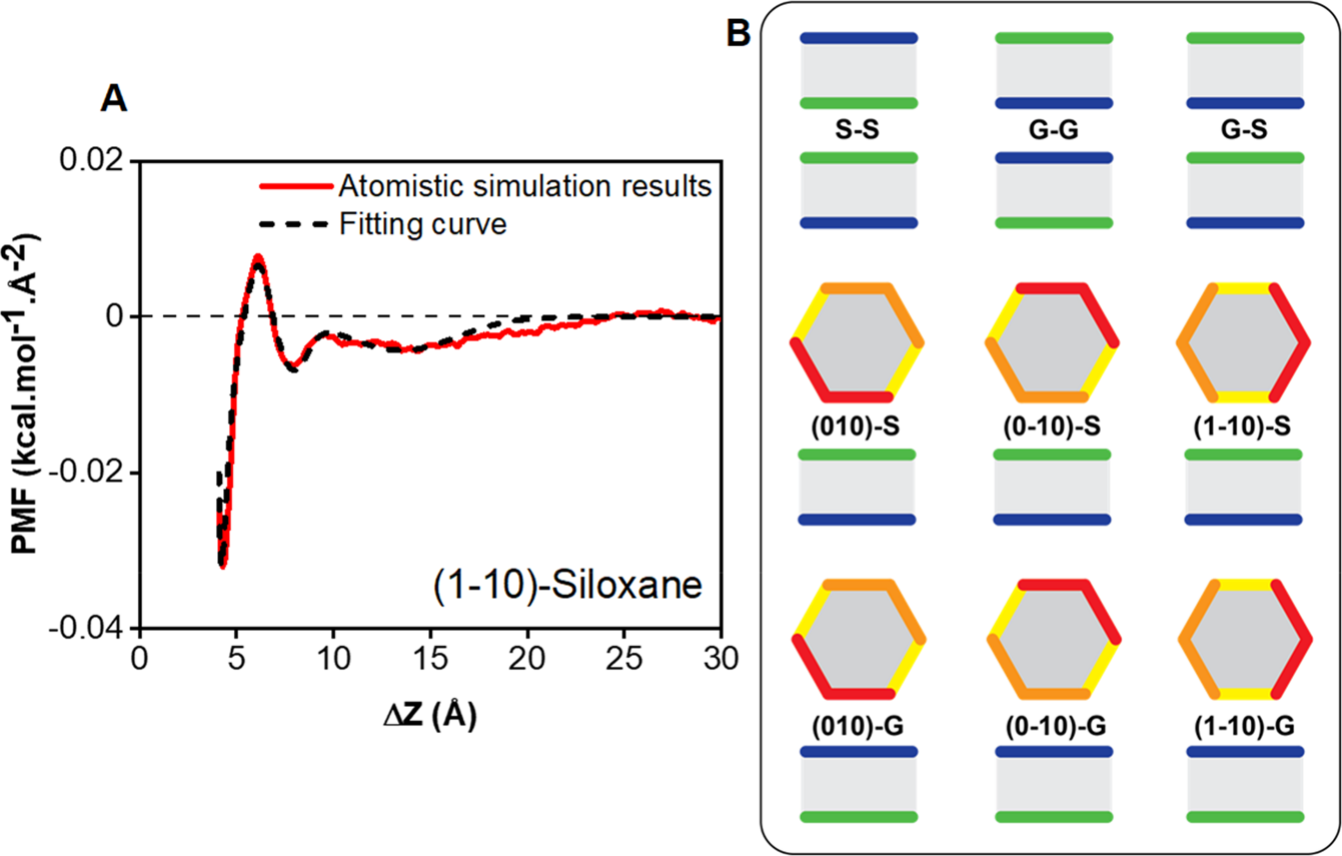
Panel A: PMF profile normalized per surface area between two kaolinite particles along their (1–10)-siloxane orientation in pure water obtained from atomistic MD simulations (red continuous line) and least-squares fitting of eq 1 (black dashed line). The atomistic results are from our prior work.^20^ The fitting parameters in eq 1 are summarized in Table S1. Panel B: schematic illustrating the possible orientations considered in the calculation of PMF profiles: gibbsite–gibbsite, gibbsite–siloxane, siloxane–siloxane, and edge–face interactions, respectively. More details can be found in our prior work.

The interactions between clay particles at the CG level were modeled by fitting the PMF profiles obtained from atomistic MD simulatons^20^ to analytical expressions obtained by modifying the semiempirical analytical models proposed by Shih et al.,^34^ Lin et al.,^35^ and Cardellini et al.^36^ Such a model was used to fit the potential of mean force per unit area, ϕ, between two clay particles separated by a distance ΔZ:
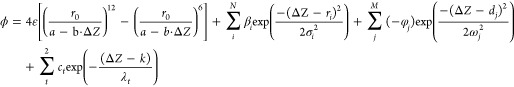
1In eq 1, ε and *r*_0_ in the first term are the parameters in the Lennard-Jones potential. The second term (*N* is the number of energy barriers) characterizes the height (β_*i*_), location (*r*_*i*_), and width of energy barriers (σ_*i*_), the third term (*M* is the number of energy wells) characterizes the depth (φ_*j*_), location (*d*_*j*_), and width of energy wells (ω_*j*_), and the last term describes the repulsive effects of an entropic nature. All parameters were determined by performing a least-squares fitting of the analytical expressions for the PMF curves for face-to-face and face-to-edge kaolinite particle interactions. In Figure 2, we show one example of the results obtained from such fitting. The parameters of eq 1 fitted to the various atomistic PMF profiles of pure water systems are listed in Table S1. The fitting parameters for PMF profiles in saline water systems are reported in Table S2. The resultant coarse-grained potentials are effectively smoothed versions of the original PMF curves, which can then be used in coarse-grained MD simulations. While tabulated versions of the original PMF curves could be used for the same purpose, the availability of the equations could facilitate the use of these results by others while also providing possible physical interpretation to the parameters shown in Tables S1 and S2 provided as Supporting Information (SI). In our approach, the CG simulations are conducted in implicit solvents, thereby allowing for significant savings of computing time.

### 2.3 Validation of the Coarse-Grained Model and Coarse-Grained Force Field

We validated the CG model by computing the interactions between two CG kaolinite nanoparticles as a function of their vertical distance (Z direction). Our approach was validated against the interaction curves obtained in Section 2.2. The overall net interaction between two particles was treated as the sum of all interactions between the constituent spheres. Because each kaolinite particle was treated as a rigid body, intraparticle interactions were excluded. The interaction energy between two particles obtained from the fitting result was assigned to each sphere representing the face and edge after scaling it according to the number of spheres found on each face or edge.

**Figure 3:**
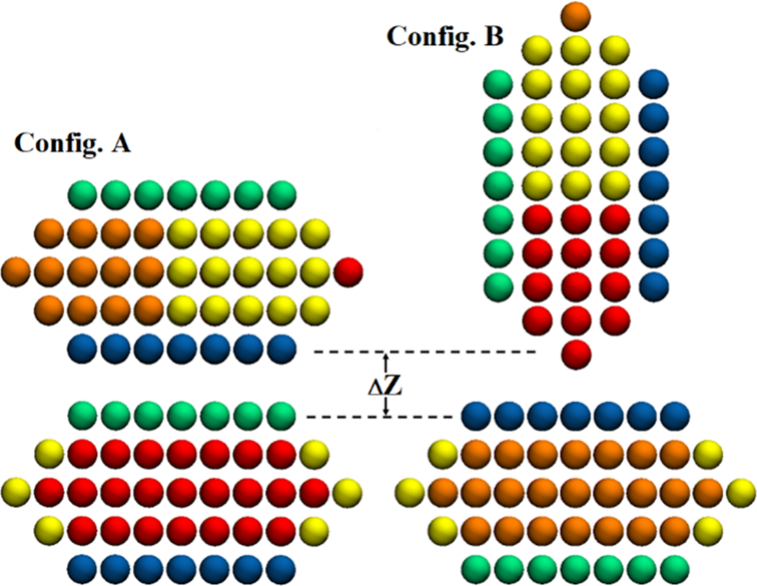
Snapshots showing relative particle orientation in configurations A and B. The color scheme is defined in Figure 1.

**Figure 4:**
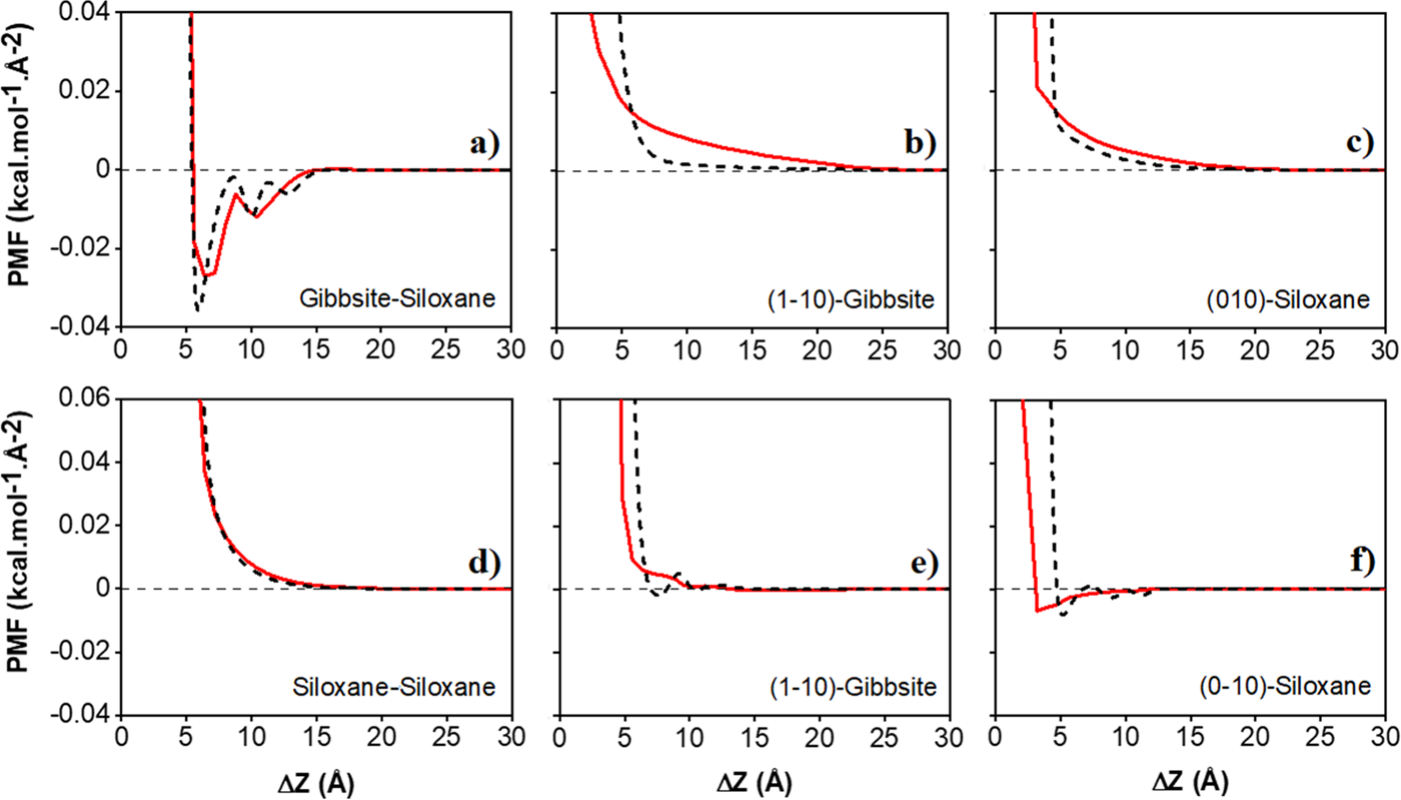
Interactions between two coarse-grained kaolinite nanostructures as a function of interparticle separation (red continuous lines) computed for varying orientations in implicit pure (a, b, c) and saline water at 1.2 M NaCl concentration (d, e, f). Please note that the relative orientation between the interacting kaolinite particles differ across the panels, as indicated by the insets. The full set of results for interactions between particles in the two solutions is reported in the SI. The results were validated against the atomistic PMF curves (shown as black dashed line). The simulations were conducted at 350 K. The relative orientations between the interacting kaolinite particles are illustrated in panel B of Figure 2.

In order to be consistent with the anisotropic interactions discovered between the atomistic clay particles in our previous study,^20^ and briefly discussed in Section 2.1, two relative configurations were considered for the kaolinite nanoparticles (Figure 3). While configuration A represents two interacting kaolinite particles parallel to each other, configuration B refers to a configuration where the top particle is oriented perpendicular to the bottom one (Figure 3). Configuration A and B explore face–face and edge–face interactions, respectively. The comparison between interaction potential results obtained for the CG model and the PMF profiles obtained from atomistic simulations at various orientations are shown in Figure 4. The results for all systems are provided in the SI (Figure S3 and S4). In our previous study, we observed distinct patterns in the PMF profiles, depending on the separation distance between the two particle surfaces. For distances beyond ∼1.5 nm, the nature of interaction, whether attractive or repulsive, is primarily governed by electrostatic forces between the solid particles. However, at shorter distances, the structural arrangements of water molecules, as well as those of hydrated ions confined between the surfaces of the two interacting particles, become a key factor in shaping the interaction profile. As observed also for interactions between solid alumina particles dispersed in aqueous electrolyte solutions,^37^ our results show that the hydration layers cause local maxima and minima in the free-energy profiles, which is the molecular phenomenon for PMF profiles such as those shown in Figure 4, which are corrugated at the subnanometer resolution. As described in our prior report,^20^ adding NaCl to the aqueous solutions has a strong effect in modulating the PMF profiles. For example, the attraction between gibbsite and siloxane surfaces becomes less pronounced because NaCl screens the interactions. Other differences can be observed in the SI. For a description of the molecular reasons responsible for these differences, the interested reader is referred to our prior manuscript.^20^ In general, our CG interaction models reproduced reasonably well the interparticle interactions in three face–face orientations in both pure water and saline solution. Although the models do not accurately reproduce all of the minute details of the PMF curves at some edge–face orientations, they are able to capture the overall attractive or repulsive nature of the interaction between nanostructures. This relatively good agreement is assumed to validate our procedure for developing the CG potential.

The resultant CG interactions were subsequently used to integrate the equations of motion within the Langevin thermostat (details below). It should be noted that in doing so, it is assumed that the free energy profiles represented by the PMF curves obtained at the atomistic level are representative of the potential interactions between particles. This approximation is likely to affect the transferability of the potentials presented here to different conditions (e.g., different temperatures and also varying concentrations of the kaolinite particles in the system). Hence, it helps to remember that the atomistic simulations and all of the CG simulations presented here were conducted at 350 K, which is representative of typical conditions encountered in geological formations.

### 2.4 Coarse-Grained MD Simulation Details

All coarse-grained simulations were carried out using the open-source LAMMPS code, version released on Sep 29, 2021.^38^ The CG simulations were performed in a cubic cell 650 Å × 650 Å × 650 Å in size using periodic boundary conditions in all three dimensions. The number of rigid hexagonal particles was changed to achieve the desired particle concentrations. The compositions of all systems considered in this study are given in Table 1.

**Table 1:** Composition of the Simulated Systems^a^

System	⧧ Particles	% V/V (Volume Fraction)
1	50	1.04
2	75	1.56
3	100	2.08

a Because the simulations are conducted in the implicit solvent approximation, once the number of particles is fixed, the particle volume fraction by volume is determined by adjusting the volume of the simulation box.

We conducted simulations in the NVE ensemble, where the number of particles (N), the simulation volume (V), and the energy (E) are kept constant. The fitting results to the simulated PMF data, as reported in Section 2.2, were translated into tabular potentials for simulation input to describe the particle interactions.

The Brownian motion of the clay particles was integrated by NVE Langevin dynamics, which is described by the Langevin equation.^39^ The Langevin equation can be considered an extension of Newton’s law of motion with the addition of frictional and random forces:
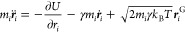
2In eq 2, *m*_*i*_ and *r*_*i*_ are the mass and position of particle *i* at time *t*, *U* is an interatomic potential as a function of particle positions, *k*_B_ is the Boltzmann constant, *T* is the desired temperature, *r*_*i*_^G^ is the Gaussian distributed noise, and γ is the Langevin friction coefficient (Section 2.4.1).

The velocity–Verlet algorithm was used to update the position and velocity of the particles with a time step of 10 fs. The temperature of all systems was kept constant at 350 K using the Langevin thermostat,^40^ with a relaxation time of 1 ps. This is the temperature at which the atomistic PMF profiles were obtained. The coarse-grained simulations were terminated when the formation of clusters appeared to be stable within a simulated time of 18 μs. For the simulations representing particles in brines, the size of the aggregate was found to oscillate during the simulations, as some aggregates grew and others broke, suggesting that equilibration was achieved. For simulations representing pure water conditions, the aggregates were found to be rather long. Although they did not break, they did not extend across the boundaries of the simulation box. To confirm the results were acceptable, we investigated the effect of simulation box size and confirmed that the results were consistent.

#### 2.4.1 Friction Coefficient for Langevin Simulations

The Langevin thermostat is commonly used to approximate solvent interactions in implicit-solvent simulations. It can reproduce the phase–space distribution in equilibrium systems with any choice of Langevin friction coefficient.^41^ However, the accuracy and efficiency of the coarse-grained simulation are controlled by the friction coefficient,^40^ as the frictional and random forces can influence particle dynamics, thus affecting transport properties.^42^ We calculated the self-diffusion coefficients of clay particles from simulations with a Langevin thermostat and different friction coefficients. We then compared them to the diffusion coefficient calculated from atomistic MD simulations in our previous study^20^ to find the correct friction value with respect to the chosen time step. To calculate the self-diffusion coefficient, we conducted simulations in which one kaolinite particle was placed in a cubic box 650 Å × 650 Å × 650 Å in size, with periodic boundary conditions. The simulations were carried out at a target temperature of 350 K, with friction coefficients ranging from ∼0.14 to 1 ps^–1^. The total simulation time was 4 μs, and the last 0.1 μs of the simulations were used for production.

The self-diffusion coefficients were calculated from the mean square displacement by implementing the Einstein equation:^43^
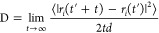
3where *r*_*i*_(*t*) and *r*_*i*_(*t*′) are the positions of particle *i* at time *t* and at the time origin *t*′, respectively, and *d* is the number of degrees of freedom. It is expected that the diffusion coefficient is proportional to the temperature of the system and inversely proportional to the particle size, as prescribed by the Stokes–Einstein relation:^44^
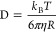
4In eq 4, *k*_B_ is Boltzmann constant, *T* is the temperature, η is the pure solvent viscosity, and *R* is the solute radius. In the previous study, we found that the bulk value of the diffusion coefficient of one kaolinite particle is similar in pure and saline water: ∼6 × 10^–11^ m^2^/s. The diameter of one atomistic kaolinite particle was ∼40 Å. Applying these values to eq 4, we obtain the diffusion coefficient of the CG particles as ∼3.43 × 10^–11^ m^2^/s. From the relation between friction and diffusion coefficients, as reported in Figure S5 of the Supporting Information, we extracted the friction coefficient for coarse-grained models as ∼0.33 ps^–1^.

### 2.5 Identification of Aggregates in the CG Simulations

For every 100 simulation steps, a cluster analysis was performed to identify and characterize aggregates of primary particles. Kaolinite primary particles were assigned as being part of an aggregate if the distance between their centers of mass was within a distance cutoff. The cutoff value is described in the results section, where a motivation for choosing said value is provided based on the simulation results. An adjacency matrix was then constructed according to this connectivity, yielding a graph representative of the state of the system. Particles aggregates were identified as the fully connected components of the graph using a depth-first search algorithm (facilitated by the NetworkX Python library^45^). To characterize the dynamic evolution of the system, we monitored the size of the four largest clusters during the simulation time as well as the number of free particles, i.e., those particles which were not connected to any other particles based on our distance criterion. Four aggregates were chosen because this number was considered large enough to provide significant statistical properties, within the constraints of our simulations. The simulations started with the particles dispersed in the simulation box. If, during the simulations, two particles are found within the cutoff distance, then they are considered to be part of an aggregate.

## 3 Results and Discussion

### 3.1 Structure of the Kaolinite Aggregates

**Figure 5:**
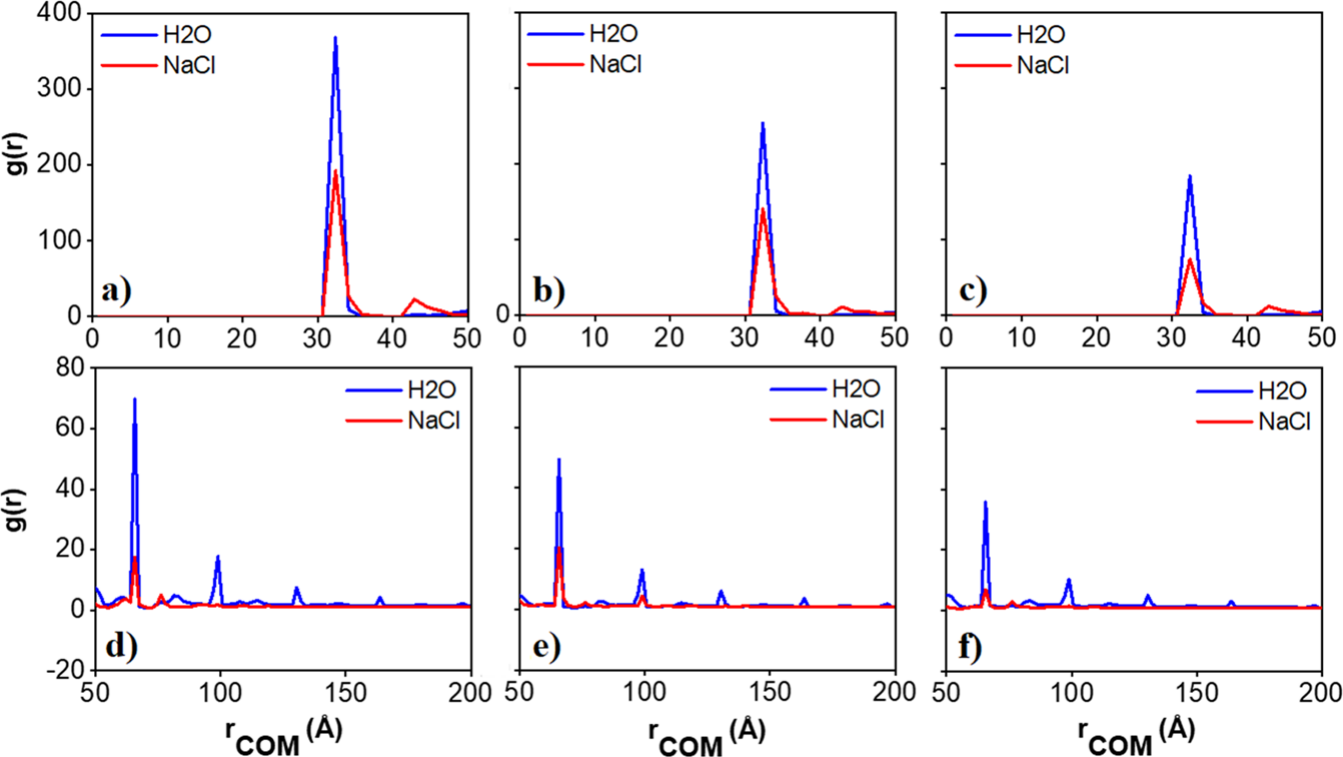
Radial distribution functions between the centers of mass of kaolinite particles calculated for the pure and saline water systems at varying kaolinite concentrations: (a and d) 1.04, (b and e) 1.56, and (c and f) 2.08% v/v. The last 3 μs of the simulations were used for data analysis. For clarity purposes, in the top panels, the results are shown for distances up to 50 Å, while in the bottom panels the results are shown for distances up to 200 Å.

Figure 5 shows the radial distribution functions (RDFs) calculated between the centers of mass of kaolinite particles in pure and saline water. The results show well-defined peaks at regular distance intervals, with the first peak being found at ∼33 Å. The distance between consecutive peaks is ∼33 Å, which approximately corresponds to the thickness of one kaolinite particle in our CG model (i.e., 28 Å when center-to-center distances between the spheres making up the nanostructures are considered). Hence, the RDF peaks represent the formation of CG aggregates with the kaolinite particles assembled with basal surfaces in parallel.

The results also show a strong effect due to the presence of NaCl salt in the aqueous system: while in pure water the peaks continue to appear at distances of ∼150 Å, when NaCl is added to the system (red curve), only the first, second, and third peaks appear in the RDF profile, while the peaks at further distances disappear. This suggests that the aggregates become smaller in saline solutions, which is expected because the PMF profiles become less attractive.^20^ While the first and third peaks reflect the face–face association, the second peak represents the association in the edge–face orientation. This is consistent with visual observation of simulation snapshots and expectations based on the PMF profiles, which are strongly anisotropic and become less attractive in the presence of NaCl. Typical aggregate structures, analyzed in detail in what follows, show that particles indeed tend to aggregate along gibbsite–siloxane and (1–10)-siloxane orientations. The observations are consistent with the PMF results obtained from atomistic MD simulations, which show that the gibbsite–siloxane orientation is the most attractive among those considered. In general, our simulations show the preferential formation of columnar structures with a few defects present. The columnar structures result from the attractive gibbsite–siloxane PMF curves, while the fact that few defects are observed is a consequence of the relatively weak edge–face PMF profiles.

Based on the RDFs describing the simulated aggregates, we define a distance criterion, which will then be used in the clustering procedure described in Sections 3.2 and 3.3. Explicitly, two particles are assumed to belong to the same aggregate if their centers of mass are less than ∼70 Å apart. We used this distance as a criterion for the analyses of aggregation.

**Figure 6:**
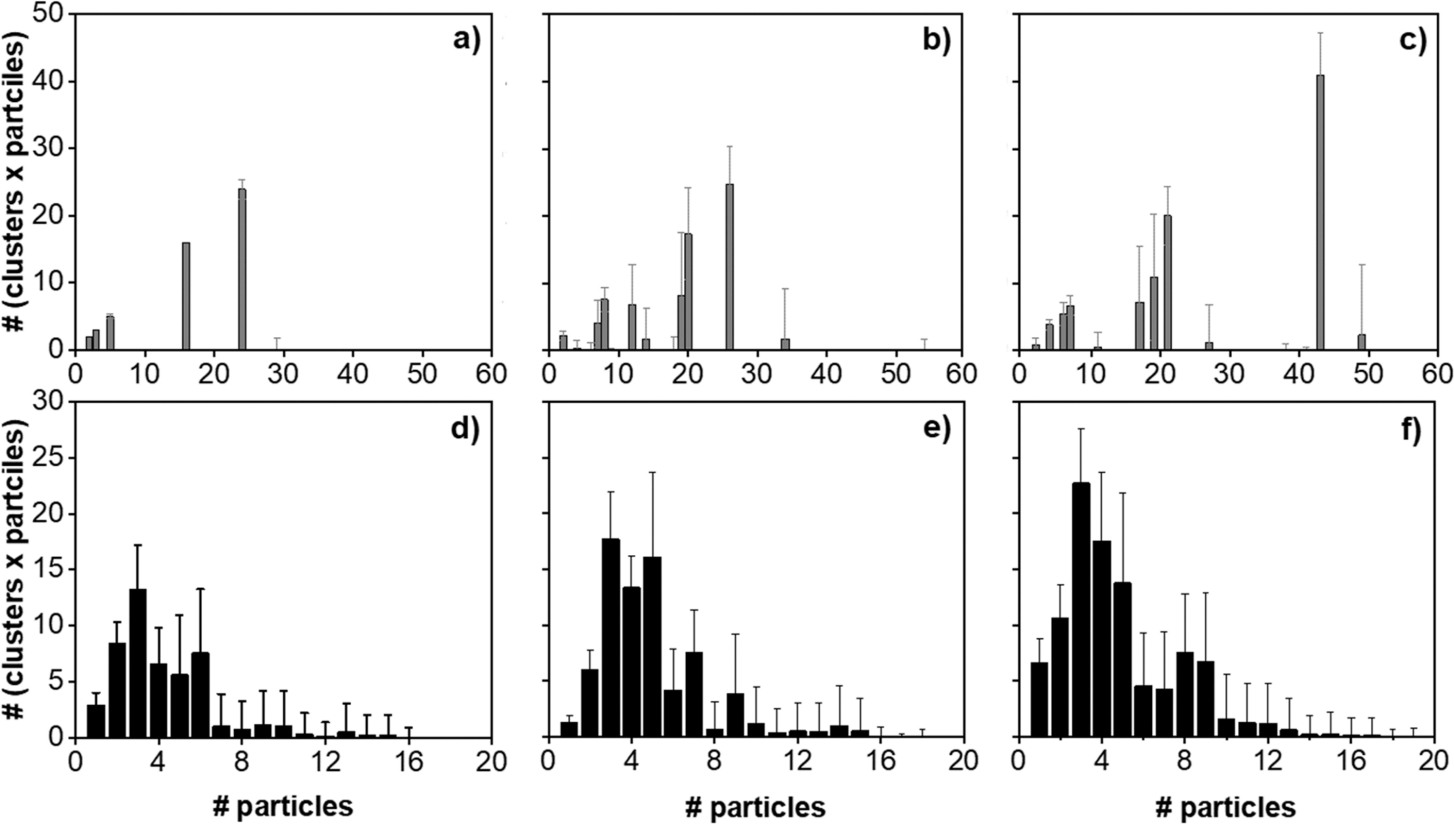
Size distribution of aggregates over the last 3 μs of simulation in pure water (a, b, c) and in 1.2 M of sodium chloride solution (d, e, f) at increasing kaolinite concentrations: (a and d) 1.04, (b and e) 1.56, and (c and f) 2.08% v/v.

To further analyze the aggregates, we investigated their size distribution over the last 3 μs of the coarse-grained simulation. The results shown in Figure 6 confirm the formation of large aggregates in pure water, although when the CG interaction potentials take into consideration the presence of NaCl in the brines, the aggregates tend to be smaller. Combined with the mean aggregate lifetime results discussed in Section 3.3, these observations suggest that in NaCl, the supra-molecular aggregates do not form clusters of size larger than the critical nuclei. On the contrary, kaolinite particles dispersed in pure water form clusters above this critical size and indeed grow to form large structures. It is remarkable that such large consequences are simply due to small changes in the PMF profiles, which were documented in our prior atomistic simulations.

### 3.2 Salt Effects on Aggregate Structure

**Figure 7:**
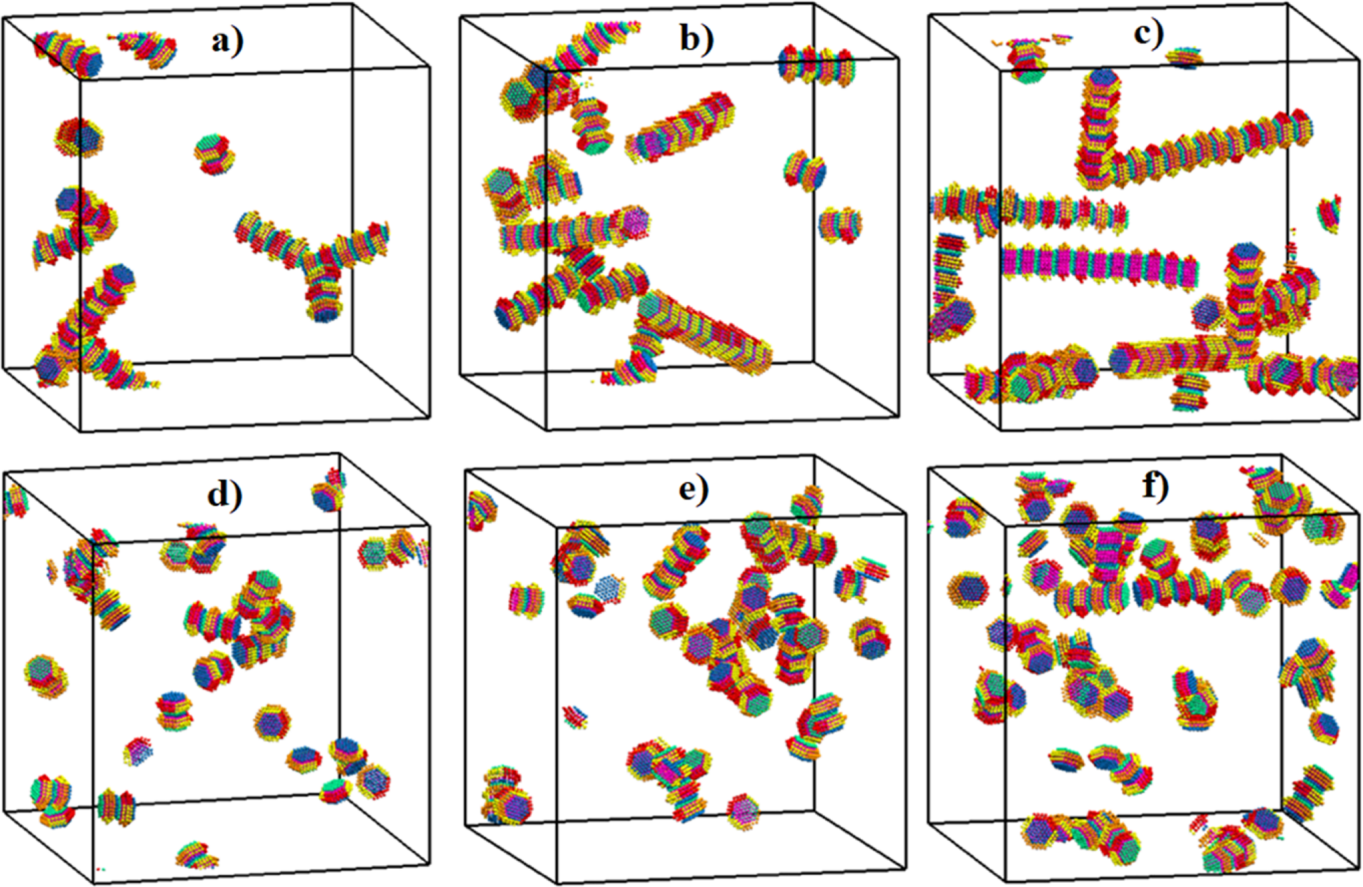
Snapshots of aggregates formed in pure water (panels a, b, c) and in aqueous systems containing NaCl ions (panels d, e, f). The results are obtained at varying kaolinite concentrations: (a and d) 1.04, (b and e) 1.56, and (c and f) 2.08% v/v. The CG simulations are conducted in implicit solvents, to speed up the calculations. The color scheme is defined in Figure 1.

**Figure 8:**
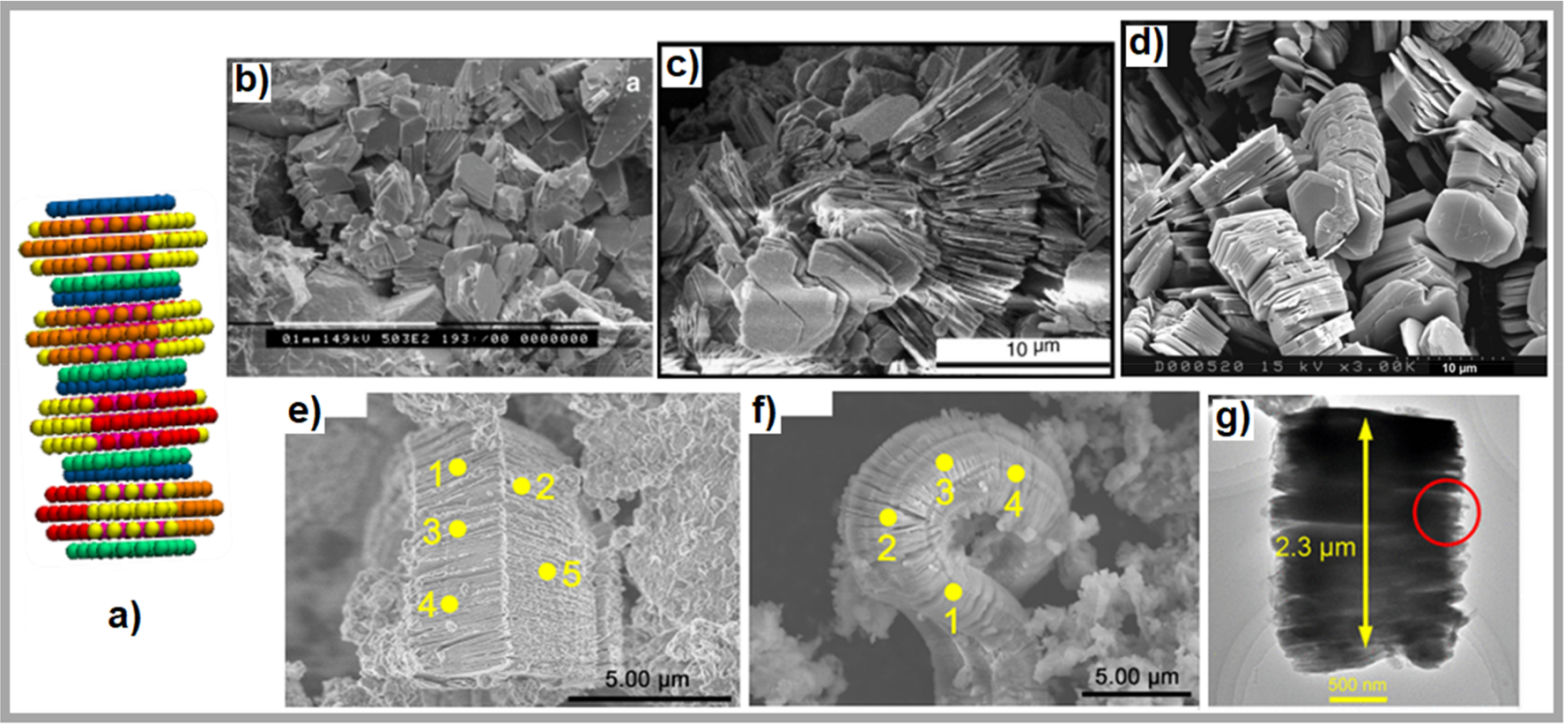
Representative configuration of particle association along face–face orientation (a). SEM image showing distribution of kaolinite booklets in one sample from North Sea reservoir sandstone (reprinted with permission from ref (46). Copyright 2014 Elsevier B.V.) (b). SEM image showing stacked kaolinite plates in the Warchha Sandstone (reprinted with permission from ref (47). Copyright 2010 Elsevier B.V.) (c). SEM image of kaolinite booklets occurring in sandstone adopted from ref (10) (d). SEM and TEM images of kaolinite aggregates with a booklet morphology (reprinted with permission from ref (48). Copyright 2022 American Chemical Society) (e, f, and g).

While the results in Section 3.1 provide an overview of the statistical features correlated with the self-assembly of kaolinite particles in aqueous dispersions, the goal of this project is to quantify the effect of NaCl on the structure of the kaolinite aggregates. The differences in PMF due to the presence of salt in water are highlighted in our prior work, and the RDF results shown above demonstrate that there is a correlation between less attractive PMFs and less structured RDFs. In this section, we quantify these differences with emphasis on visual characterization. For completeness, it is worth repeating that the CG simulations are conducted within the implicit solvent approximation, which allows us to probe relatively long phenomena with reasonable computational resources. In Figure 7, we report the configurations obtained for systems simulated at varying kaolinite concentrations, in pure water or in salty water, at the end of our simulations. In pure water, particles are found to self-assemble, yielding one-dimensional aggregates, which are longer than those formed in the presence of NaCl. Although we observe different particle associations (i.e., face–face and edge–face), face–face aggregation is dominant in both environments. The face–face structure is reminiscent of the booklet kaolinite structure observed in numerous scanning electron microscopy (SEM) images. Kaolinite often appears in sandstones as highly packed and parallelly stacked plates, in booklet or vermicular form, and as discrete aggregates within pore spaces.^10^ SEM and transmission electron microscopy (TEM) images of kaolinite aggregates with a booklet morphology are shown in Figure 8. For completeness, it should be pointed out that the experimental kaolinite platelets observed in these experiments are ∼5–10 μm in size, which is much larger than that of the particles used in our simulations. Nevertheless, the qualitative agreement between the experiments and simulations is encouraging.

It is worth noting that our simulations predict that as the kaolinite particle concentration increases in pure water, self-assembly leads to the formation of more extended aggregates, which tend to adopt a columnar structure with primary particles arranged in parallel layers. Because the height of these 1D columnar aggregates could be affected by the size of the simulation box, it is crucial to consider the impact of the simulation box size when studying the size and structure of aggregates. A discussion is presented below to clarify the influence of the simulation box size on the aggregate size.

In pure water, besides the formation of long columnar structures as the concentration of particles increases, we observe associations in which particles attach to the side of the columnar aggregates adopting edge–face orientations. This phenomenon is less prominent in the presence of NaCl. In saline solutions, the main structural configurations observed are somewhat distorted edge–face and face–face booklet associations in which the particles associate forming various angles with respect to each other.

**Figure 9:**
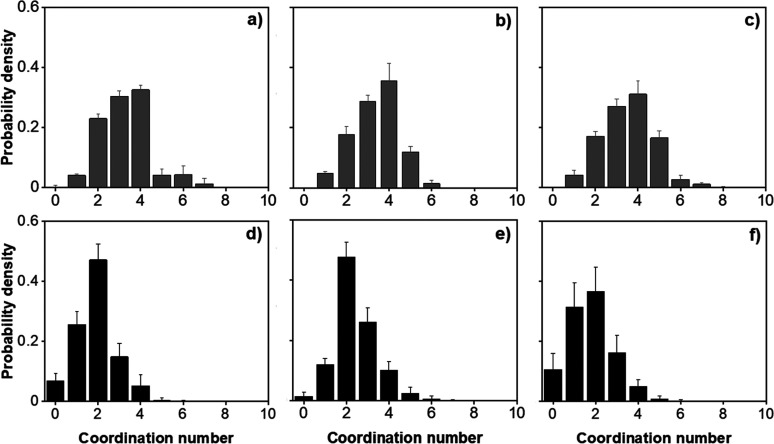
Probability of coordination numbers for individual particles in pure water (a, b, c) and in 1.2 M of sodium chloride solution (d, e, f) at increasing kaolinite concentrations: (a and d) 1.04, (b and e) 1.56, and (c and f) 2.08% v/v. The last 6 μs of the simulations were used for data analysis.

To quantify the number of defects present in the simulated aggregates, we calculated the distribution of coordination numbers for individual particles in pure water and saline environments over the final 6 μs of simulation trajectories. As shown in Figure 9, in pure water, most particles are surrounded by more than one neighboring particle, typically between two and four, suggesting that only few defects are present. On the contrary, the probability of observing more than two particles around one particle in the CG simulations parametrized to represent pure water conditions is substantial; it implies that in this system, kaolinite particles can form aggregates with structures beyond the columnar arrangement.

### 3.3 Dynamical Properties of the Self-Assembled Aggregates

**Figure 10:**
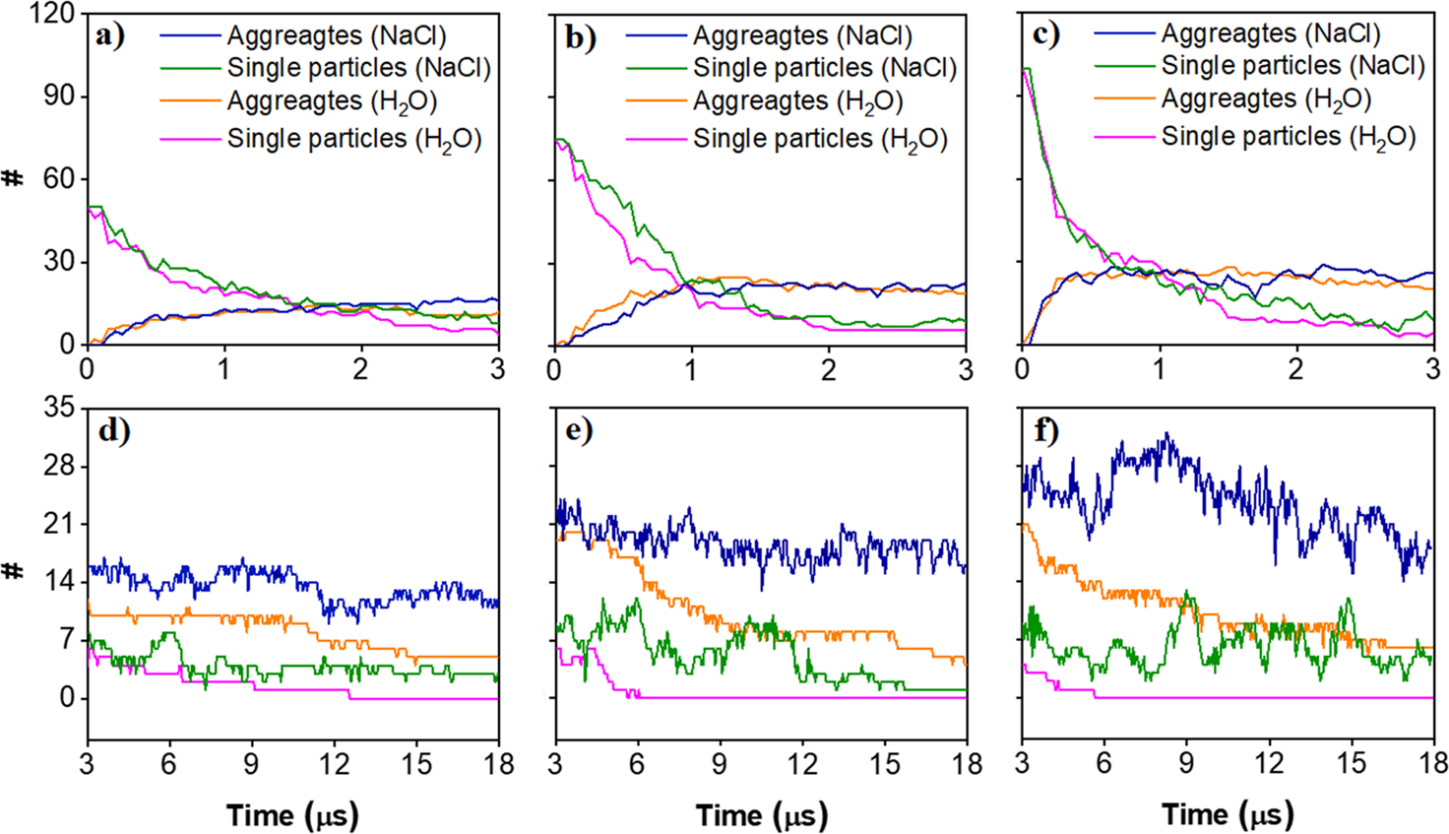
Time evolution of the number of aggregates in pure water and in 1.2 M of sodium chloride solution at increasing concentrations of kaolinite particles: (a and d) 1.04, (b and e) 1.56, and (c and f) 2.08% v/v. For clarity purposes, we present results in two plots: in the top panels, the results obtained within the first 3 μs of the simulations are highlighted, while in the bottom panels, we show the results up to 18 μs.

To complement and better quantify the structural information extracted from simulation snapshots, such as those shown in Figure 7, we calculated the evolution of the number of aggregates with time for pure and saline water systems at varying concentrations of kaolinite. It should be remembered that the simulations are conducted in implicit solvent model, which speeds up the dynamics of the system. With this limitation in mind, our simulation results are displayed in Figure 10. In saline (NaCl) solutions, we observe more aggregates formed during the simulation trajectory than those in pure water. While no individual particle is found in pure water systems at the end of simulations, 2–3 isolated kaolinite particles are observed in the 1.2 M sodium chloride solution. Also, we notice a rapid increase in the number of aggregates in the first ∼2 μs, which converges over the remainder of simulations. The number of aggregates in pure water reaches a plateau at ∼12 μs, while this happens much faster in the presence of ions. An increase in the number of aggregates with an increase in the kaolinite concentration from 1.04 to 1.56% v/v is observed in pure water systems. However, increasing the concentration up to 2.04% v/v does not seem to produce more aggregates. Meanwhile, the number of aggregates formed in saline solution increases as the kaolinite concentration increases from 1.04 to 2.08% v/v.

To complement the results in Figure 10, we monitored the size of the four largest aggregates during the simulation trajectory. These aggregates were chosen because, since they reached the largest dimensions in our simulations, it was assumed that by analyzing their trajectory, we could identify, qualitatively, the mechanisms involved in aggregates growth. For brevity, the simulation results are reported in the SI (Figure S6). Our results show that the size of the aggregates formed in pure water is much larger than that in saline solution, which is consistent with observations from Figure 6, Figure 7, and Figure 10. For example, one of the largest aggregates formed in pure water at the highest kaolinite concentration contains 43 particles, which is approximately triple the average size of the aggregates in 1.2 M NaCl solution (∼14 particles; see Figure 6). As the concentration increases up to 2.08% v/v in pure water, the increase in aggregate size is more pronounced than that in an aqueous NaCl solution. The size of aggregates formed in saline solution does not significantly change with increasing concentration of kaolinite particles. Due to an increase in the size of aggregates in pure water, we conducted additional simulations to investigate the possible effects of the simulation box size on aggregate size. We report in the SI details regarding these simulations (see Table S3 and Figure S7). In both simulated environments, the results show no significant effects of box size on the size of aggregates.

Results presented in the SI (Figure S7) also show the time-dependent behavior of aggregate breakdown and coalescence during our simulations. In all systems, the association and dissociation of aggregates are observed continuously throughout the simulation, but they tend to slow down after approximately 15 μs in pure water. Furthermore, the results suggest that the larger aggregates tend to break down into smaller ones while the smaller aggregates tend to merge into larger ones over time. The time-dependent behavior of aggregate breakdown and association reveals that the aggregation process is not static but exhibits dynamic changes in the aggregate size distribution throughout the simulation.

**Figure 11:**
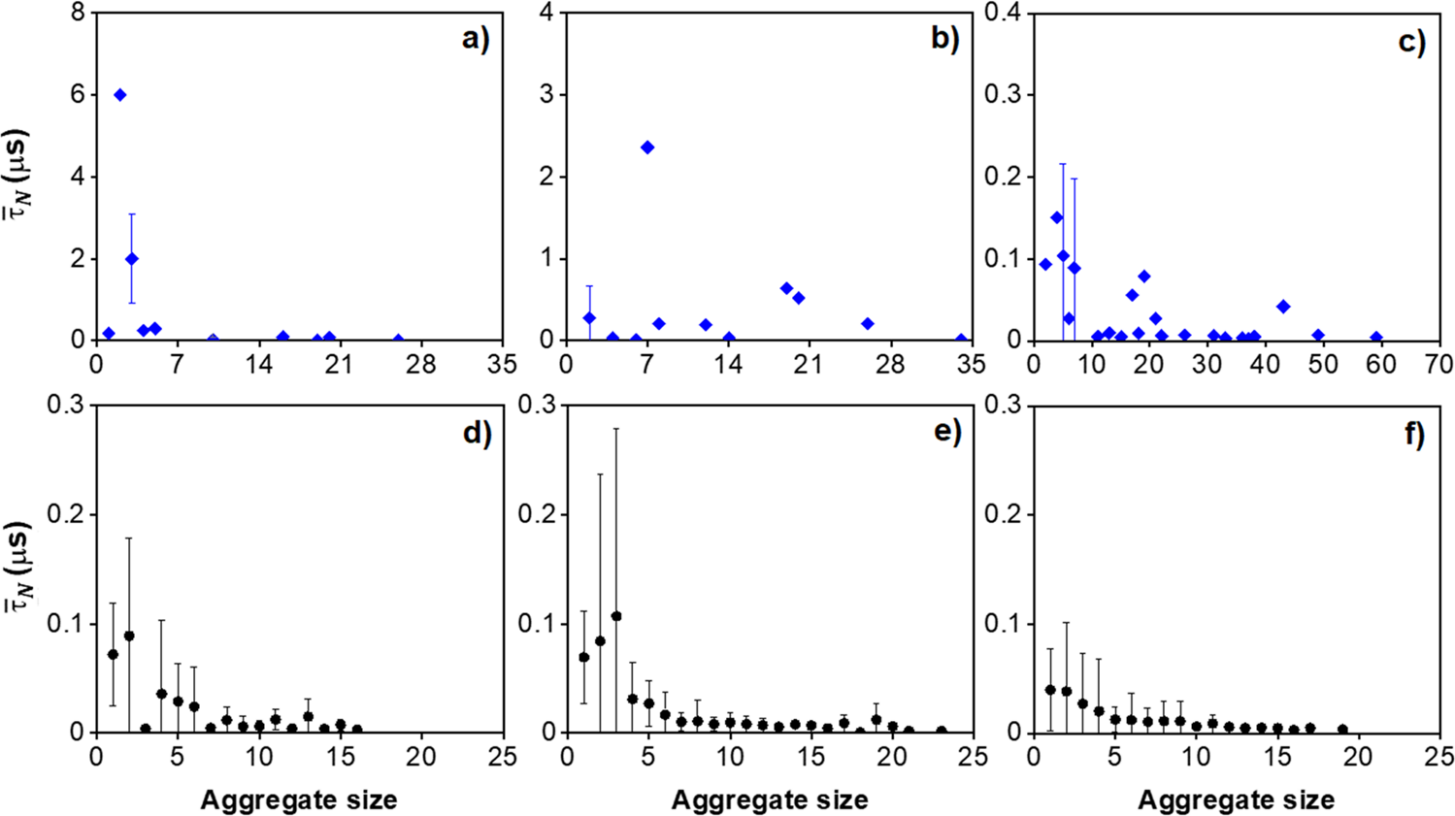
Mean aggregate lifetime as a function of the aggregation size. Data points represent measured lifetimes for aggregates simulated in CG conditions representative of pure water (a, b, c) and of 1.2 M NaCl aqueous solution (d, e, f) at increasing kaolinite concentrations: (a and d) 1.04, (b and e) 1.56, and (c and f) 2.08% v/v. The last 6 μs of the simulations were used for data analysis. Error bars represent the standard error from the mean.

To measure the longevity of aggregates formed at equilibrium, we report in Figure 11 the mean aggregate lifetime τ̅_*N*_ as a function of aggregate size in pure water and saline solution systems. The lifetime of an aggregate is defined as the duration during which it remains a connected cluster of the same kaolinite primary particles. To define the birth and death of an aggregate, one can consider the frames of observation in which these events occur. The time of birth of an aggregate is defined as the first frame in which the particles come together through a binding or fragmentation event. The instant when the death of an aggregate occurs instead is defined as the frame in which the particles that were originally part of the aggregate undergo a new binding or fragmentation event, causing the aggregate to break apart. To determine the mean *N*-mer lifetime, we calculated the lifetimes of all *N*-mers observed in the simulation trajectory and then averaged them. We excluded any aggregate with lifetime shorter than 3 ns to avoid artificially lowering the mean lifetime due to transient rebinding events.

Additionally, we did not include in our analysis those aggregates that appeared in the first or last frames of the simulations, as the timing of their formation or dissolution was not well quantified. The details of the approach for measuring the mean time between association and dissociation events can be found in the study reported by Wang et al.^49^ The results show that the lifetime of aggregates simulated in implicit solvents that account for the presence of ions is shorter than that obtained in the implicit solvents that represent pure water. For saline solutions, we also observe a decrease in the lifetime with aggregate size for the saline solution systems. These results are useful for developing a quantitative understanding of the aggregation process for kaolinite particles in aqueous systems. They also confirm that the simulations were long enough to observe formation, growth, and also dissociation of the aggregates observed during the simulations.

## 4 CONCLUDING REMARKS

In this work, we have developed a multiscale computational approach to deliver a molecular-level understanding of the mechanisms responsible for the agglomeration of kaolinite particles and then incorporate such information into a coarse-grained model. Our CG kaolinite nanostructure was built from five types of spheres to model various anisotropic interactions between the facets of individual kaolinite platelets. The underlying model allows one to explicitly account for the anisotropy of particle–particle interactions that was revealed by atomistic simulations in explicit solvent, as well as for the geometrical anisotropy of the particles themselves. Conducting the simulations in an implicit solvent allows us to achieve relatively long simulation times. This approach enables us to directly observe the formation of different types of associated structures compared to those described in previous numerical studies conducted by implementing various interaction laws between coarse-grained platelets.^31,33^ Favorable comparison to experiments, albeit qualitative, suggests that the model developed is able to reveal realistic insights.

The simulations presented herein were conducted with various concentrations of kaolinite particles in systems representative of pure water and 1.2 M NaCl brine. The CG model can be implemented to study the effect of particle size on the aggregation mechanism. Preliminary results along these lines are included in the SI. The kinetics of agglomeration in our coarse-grained model can also be followed, although it is expected to be faster than reality due to the reduced degrees of freedom typical of coarse-grained simulations, especially when conducted in implicit solvents. The model allows us to directly correlate changes in the potential of mean forces to differences observed in the structure of particle agglomerates and their mechanism of growth. In particular, because, in the absence of salt, PMF profiles between gibbsite and siloxane surfaces are significantly attractive, particles tend to aggregate into rather long columnar structures, while the presence of salt weakens the attractive PMF profiles, leading to shorter structures formed by the kaolinite particles. When these aggregates become too long, they break because of a competition between entropic and enthalpic effects.

In general, aggregates obtained from our CG model have more complex morphologies that were not noticeable in previous numerical studies.^31,33^ In particular, our simulations provide evidence of defects, with the results being dependent on the presence of salt in the aqueous medium. Further, the results for the mean aggregate lifetime, combined with those for the size distribution of aggregates, suggest that the kaolinite particles form large structures characterized by long lifetimes in pure water, while NaCl brines maintain the nuclei at subcritical size, a fundamental insight useful for predicting crystallization and growth in complex systems. The results of this modeling approach could, in subsequent developments, be integrated as input parameters for fluid dynamics models to predict permeability, fines migration, and pore blocking in a variety of applications ranging from materials engineering to catalysis, from energy production to carbon sequestration.

## Supporting Information Available

Fitting parameters for PMF profiles in pure and saline water systems, simulated PMF profiles between two kaolinite palettes in both pure and saline water, along with the fitting results, interactions between two coarse-grained particles in pure and saline water systems validated against fitting PMF profiles, the relationship between diffusion and friction coefficients, the time evolution of the size of the four largest aggregates formed at varying kaolinite concentrations, simulation details and results regarding the effects of simulation box size on aggregate size, as well as simulation details and preliminary results obtained from simulations involving large CG particles (PDF)
