# Supplementary material for: Mesoscale Simulations Reveal How Salt Influences Clay Particles Agglomeration in Aqueous Dispersions
Source: J Chem Theory Comput. 2023 Nov 2;20(4):1612–24. doi: 10.1021/acs.jctc.3c00719 (PMC10902848; doi:10.1021/acs.jctc.3c00719)
Supplement: Supplementary file 1 — ct3c00719_si_001.pdf [file ct3c00719_si_001.pdf]

Supporting Information: Mesoscale Simulations Reveal How Salt Influences Clay Particles Agglomeration in Aqueous Dispersions

Tran Thi Bao Le,[1] Aaron R Finney,[1] Andrea Zen,[2] Tai Bui,[3] Weparn J. Tay,[3] Kuhan Chellappah,[3] Matteo Salvalaglio,[1] Angelos Michaelides,[4] Alberto Striolo[1,5]

[1]*Department of Chemical Engineering, University College London, WC1E 7JE, London, United Kingdom*

[2]*Dipartimento di Fisica Ettore Pancini, Università di Napoli Federico II, Monte S. Angelo, I-80126 Napoli, Italy*

[3]*BP Exploration Operating Co. Ltd, Chertsey Road, Sunbury-on-Thames TW16 7LN, United Kingdom*

[4]*Yusuf Hamied Department of Chemistry, University of Cambridge, Lensfield Road, Cambridge CB2 1EW, United Kingdom*

[5]*School of Sustainable Chemical, Biological and Materials Engineering, The University of Oklahoma, OK 73019, United States*

* Author to Whom all correspondence should be addressed: a.striolo@ucl.ac.uk

In **Table S1**, we report the parameters of **Eq. 1** fitted to the various atomistic PMF profiles of pure water systems.

In **Table S2**, we report the parameters of **Eq. 1** fitted to the various atomistic PMF profiles of saline water systems.

In **Figure S1**, we show the simulated PMF profiles between two kaolinite palettes in pure water systems and the fitting results to the simulated PMF data.

In **Figure S2**, we show the simulated PMF profiles between two kaolinite palettes in 1.2 M of sodium chloride solution and the fitting results to the simulated PMF data.

In **Figure S3**, we present the interactions between two coarse-grained particles as a function of interparticle separation in pure water. The results were validated against the fitting PMF profiles.

In **Figure S4**, we present the interactions between two coarse-grained particles as a function of interparticle separation in saline water. The results were validated against the fitting PMF profiles.

In **Figure S5**, we report the relation between diffusion and friction coefficients. Because diffusion coefficient of kaolinite particle in both pure water and saline solution appear to be the same, the simulations were conducted only for the saline water systems.

In **Figure S6**, we present the time evolution of the size of the four largest aggregates formed throughout the simulation at varying kaolinite concentrations.

In **Table S3**, we report the system compositions considered to investigate the effects of simulation box on the aggregate size. The simulations were performed within the Langevin formalism in the NVE ensemble at 350 K for 18 μs. In those simulations, the kaolinite concentration was kept constant.

In **Figure S7**, we present the time evolution of the size of the four largest aggregates formed throughout the simulation at varying simulation boxes.

In **Figure S8**, we present preliminary results obtained from simulations of large CG particles. The CG simulations were performed by placing 50 particles in a cubic cell 650 Å × 650 Å × 650 Å in size, using periodic boundary conditions in all three dimensions. with periodic boundary conditions applied in all three dimensions. The resulting concentration corresponds to approximately 3.18% v/v (volume fraction). The simulations were conducted in the NVE ensemble at a temperature of 350 K, employing the Langevin thermostat.

Table S1. Summary of the fitting parameters in **Eq. 1** for PMF profiles obtained from pure water systems.

	**G-G**	**G-S**	**S-S**	**(010)-G**	**(0-10)-G**	**(1-10)-G**	**(010)-S**	**(0-10)-S**	**(1-10)-S**
**ε**	-	0.036	-	0.029	-	-	-	0.003	0.032
**r_0**	-	6.215	-	5.043	-	-	-	4.583	4.289
**a**	-	1.920	-	9.278	-	-	-	14.924	6.541
**b**	-	1.495	-	3.000	-	-	-	4.473	2.682
**β_1**	0.039	0.003	-	0.004	-	-	-	0.009	0.008
**r_1**	5.172	8.368	-	6.489	-	-	-	5.057	6.116
**σ_1**	0.357	1.166	-	0.210	-	-	-	0.086	0.530
**β_2**	0.058	-	-	0.009	-	-	-	0.012	-
**r_2**	7.266	-	-	8.031	-	-	-	6.860	-
**σ_2**	0.895	-	-	0.481	-	-	-	0.910	-
**β_3**	0.018	-	-	0.003	-	-	-	0.006	-
**r_3**	10.404	-	-	10.442	-	-	-	10.180	-
**σ_3**	0.436	-	-	0.530	-	-	-	1.331	-
**β_4**	0.007	-	-	0.003	-	-	-	-	-
**r_4**	12.884	-	-	12.755	-	-	-	-	-
**σ_4**	3.099	-	-	0.941	-	-	-	-	-
**φ_1**	-	0.011	-	$0.051\text{x}10^{-2}$	-	-	-	-	0.006
**d_1**	-	9.983	-	9.381	-	-	-	-	7.943
**ω_1**	-	0.573	-	0.348	-	-	-	-	0.719
**φ_2**	-	0.006	-	-	-	-	-	-	0.004
**d_2**	-	12.782	-	-	-	-	-	-	13.521
**ω_2**	-	1.002	-	-	-	-	-	-	2.805
**φ_3**	-	-	-	-	-	-	-	-	-
**d_3**	-	-	-	-	-	-	-	-	-

**ω_3**	-	-	-	-	-	-	-	-	-
**c_1**	-	-	0.141	-	2.030	4.605	27.971	-	-
**λ_1**	-	-	4.370	-	0.084	0.853	0.153	-	-
**c_2**	-	-	120.164	-	0.07	0.005	0.017	-	-
**λ_2**	-	-	0.236	-	2.973	7.660	3.727	-	-
**k**	-	-	4.465	-	1.280	0.671	3.274	-	-

Table S2. Summary of the fitting parameters in **Eq. 1** for PMF profiles obtained from saline water systems.

	**G-G**	**G-S**	**S-S**	**(010)-G**	**(0-10)-G**	**(1-10)-G**	**(010)-S**	**(0-10)-S**	**(1-10)-S**
**ε**	0.025	0.033	-	0.017	0.009	0.002	0.013	0.011	0.024
**r_o**	5.880	6.330	-	5.027	4.892	7.551	4.698	5.380	4.480
**a**	6.168	6.050	-	41	13.844	0	2.607	0.352	1.432
**b**	2.188	2.105	-	9.273	4.052	1.078	1.642	1.227	1.459
**β_1**	0.018	0.004	-	0.011	0.006	0.006	-	0.004	0.007
**r_1**	7.780	8.560	-	5.502	5.748	9.097	-	6.645	5.35
**σ_1**	0.508	0.546	-	0.156	0.320	0.578	-	1.434	0.355
**β_2**	0.003	0.067×10^{-2}	-	0.009	0.006	0.002	-	-	-
**r_2**	10.699	11.470	-	6.262	7.843	11.996	-	-	-
**σ_2**	0.410	0.293	-	0.285	0.369	1.301	-	-	-
**β_3**	-	-	-	0.522×10^{-2}	0.002	-	-	-	-
**r_3**	-	-	-	7.972	10.413	-	-	-	-
**σ_3**	-	-	-	0.293	0.393	-	-	-	-
**φ_1**	0.009	0.005	-	0.247×10^{-2}	0.003	0.056×10^{-2}	0.008	0.003	0.011
**d_1**	9.190	10.110	-	7.212	6.986	10.450	7.450	8.790	7.37
**ω_1**	0.465	0.334	-	0.208	0.420	0.443	0.366	0.673	0.621
**φ_2**	-	0.144×10^{-2}	-	0.002	0.002	-	0.005	0.002	0.004
**d_2**	-	12.930	-	9.302	9.176	-	9.158	11.240	10.360
**ω_2**	-	0.454	-	0.524	0.389	-	1.005	0.500	1.551
**φ_3**	-	-	-	-	0.051×10^{-2}	-	-	-	-
**d_3**	-	-	-	-	11.937	-	-	-	-
**ω_3**	-	-	-	-	0.314	-	-	-	-
**c_1**	-	-	412.638	-	-	-	-	-	-
**λ_1**	-	-	0.435	-	-	-	-	-	-
**c_2**	-	-	0.235	-	-	-	-	-	-
**λ_2**	-	-	2.170	-	-	-	-	-	-
**k**	-	-	2.076	-	-	-	-	-	-

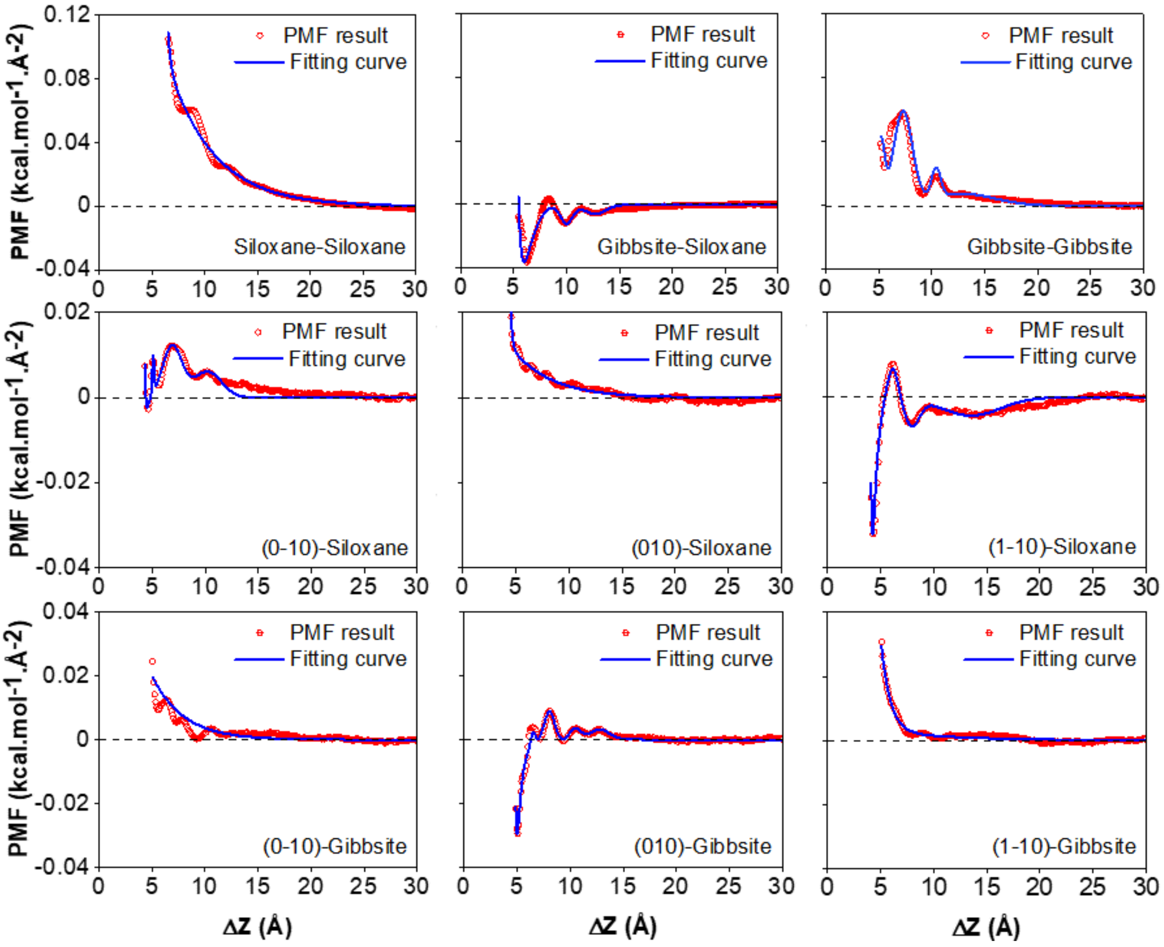

Figure S1. Potential of mean force per surface area between two clay particles in pure water obtained from atomistic MD simulations (red circles) and the fitting results.

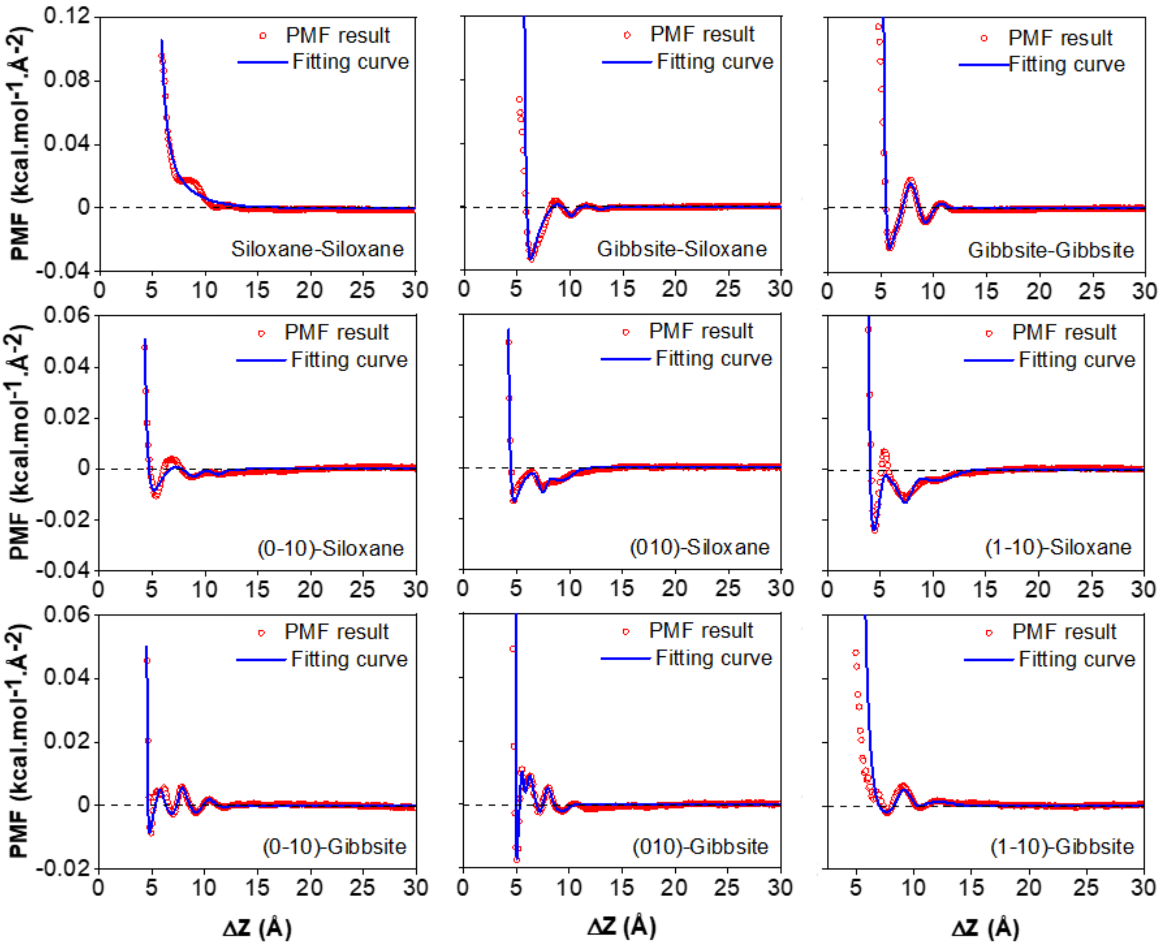

Figure S2. Potential of mean force per surface area between two clay particles in 1.2 m NaCl solution obtained from atomistic MD simulations (red circles) and the fitting results.

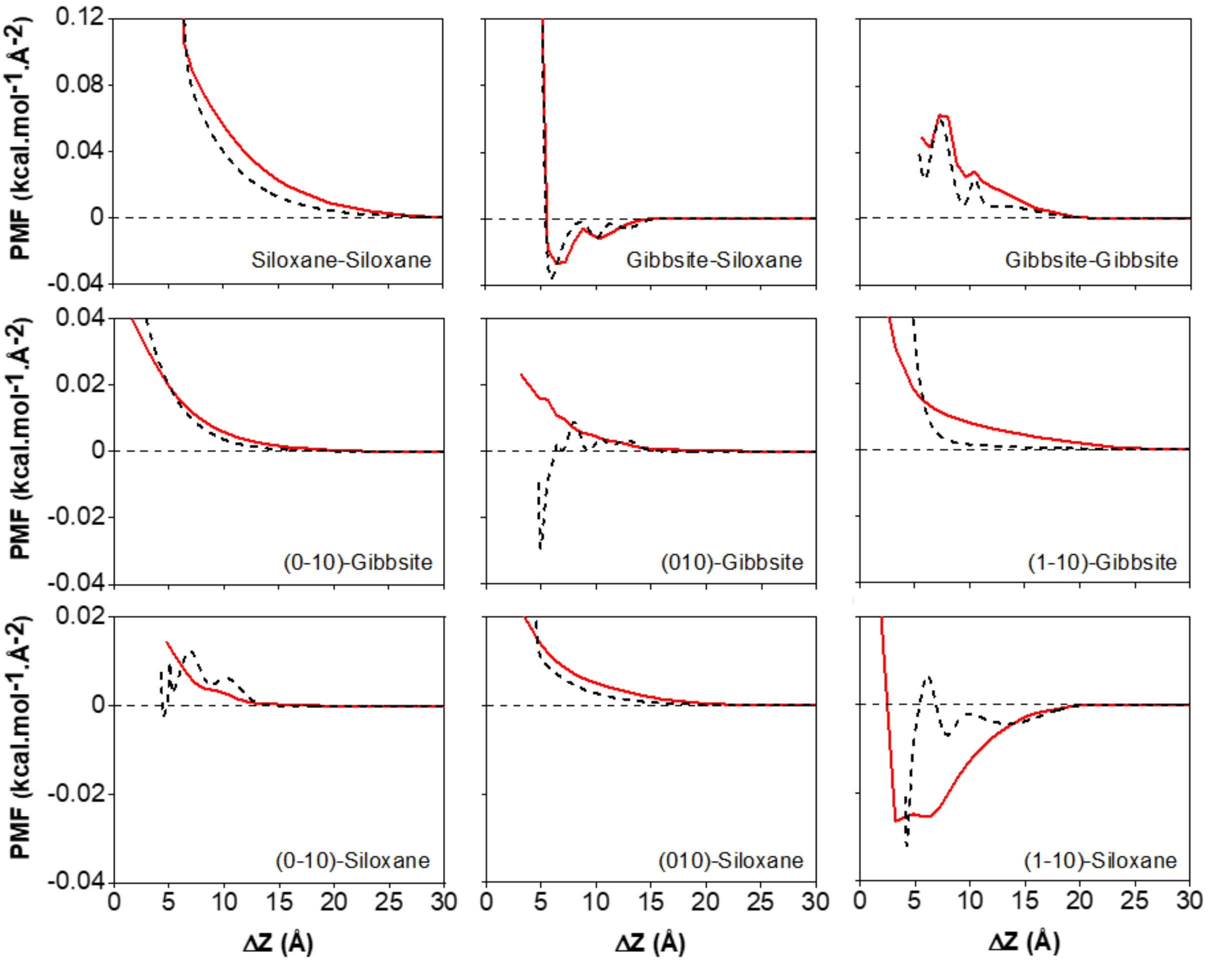

Figure S3. Interactions between two coarse-grained kaolinite nanostructures as a function of interparticle separation (red continuous lines) computed for varying orientations in implicit pure water. The results were validated against the atomistic PMF curves (shown as black dashed line).

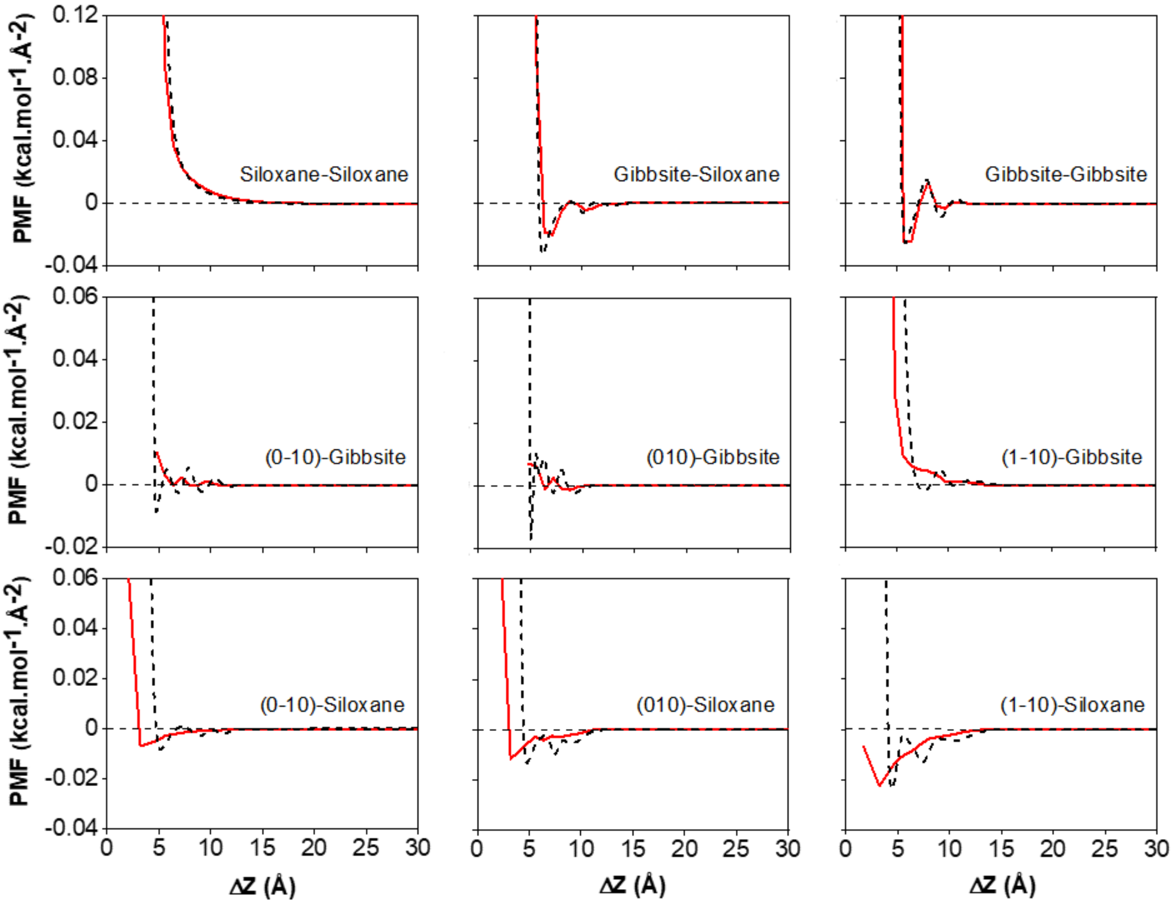

Figure S4. Interactions between two coarse-grained kaolinite nanostructures as a function of interparticle separation (red continuous lines) computed for varying orientations in saline water at 1.2 M NaCl concentration. The results were validated against the atomistic PMF curves (shown as black dashed line).

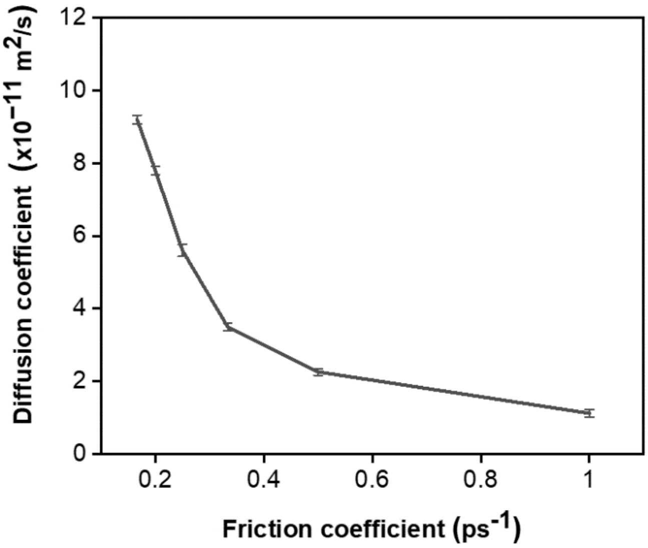

Figure S5. The relationship between friction and diffusion coefficient. The simulations were conducted in 1.2 M of sodium chloride solution.

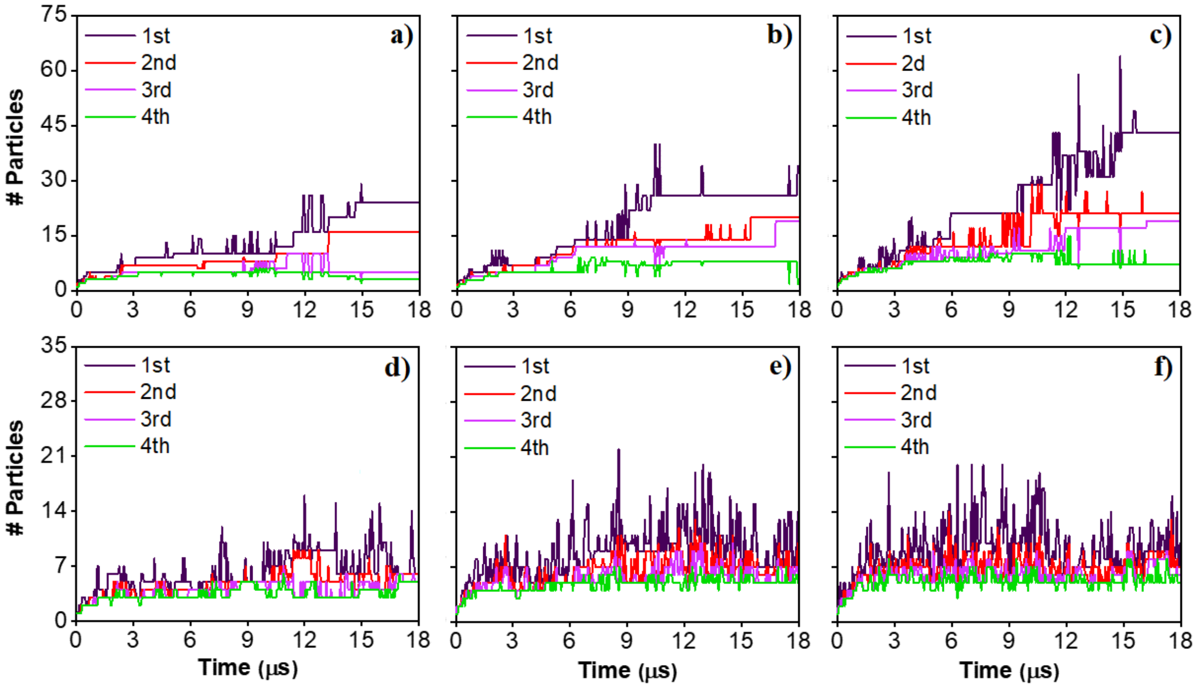

Figure S6. Time evolution of the size of the four largest clusters from simulations conducted in pure water (a, b, c) and in 1.2 M NaCl brine (d, e, f) at increasing kaolinite concentrations (a & d) 1.04, (b & e) 1.56, and (c & f) 2.08 % v/v.

Table S3. Composition of systems simulated to investigate the simulation box size effects on aggregate size.

**# Particles**	**18**	**50**	**101**
**Volume (Å^3)**	450 x 450 x 450	650 x 650 x 650	800 x 800 x 800
**% v/v**	~ 1.04	~ 1.04	~ 1.04

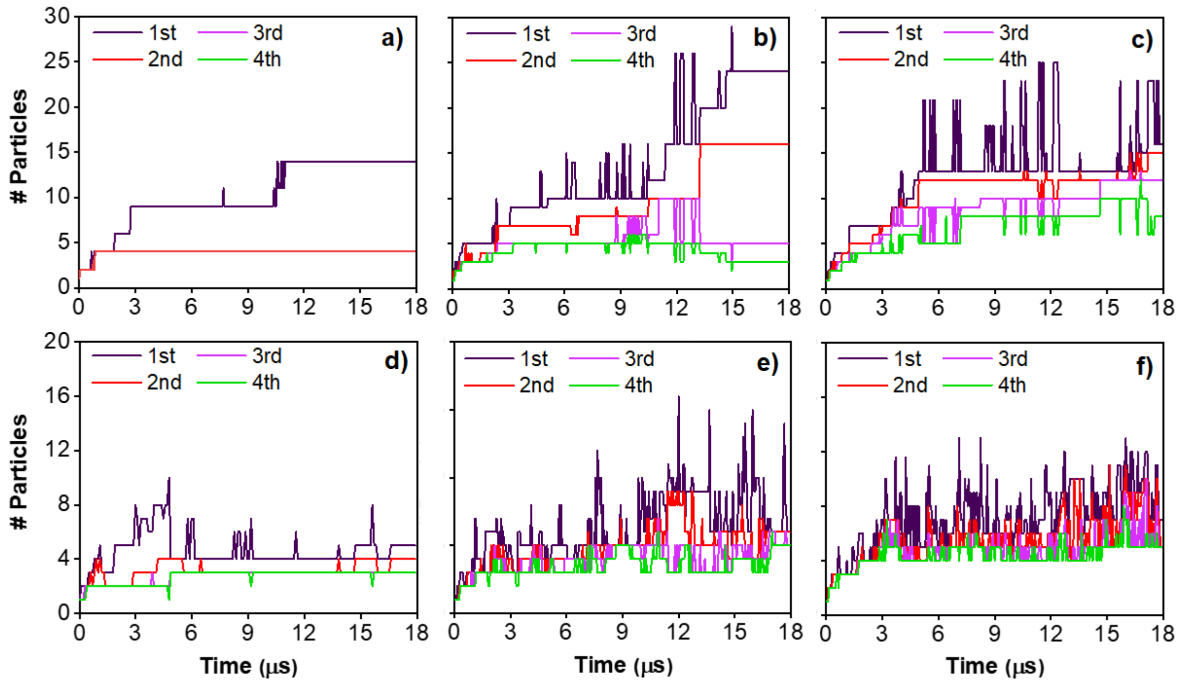

Figure S7. Time evolution of the size of the four largest clusters from simulations conducted in pure water (a, b, c) and in 1.2 M NaCl brine (d, e, f) at increasing simulation box (a & d) 450 x 450 x 450 Å^3, (b & e) 650 x 650 x 650 Å^3, and (c & f) 800 x 800 x 800 Å^3.

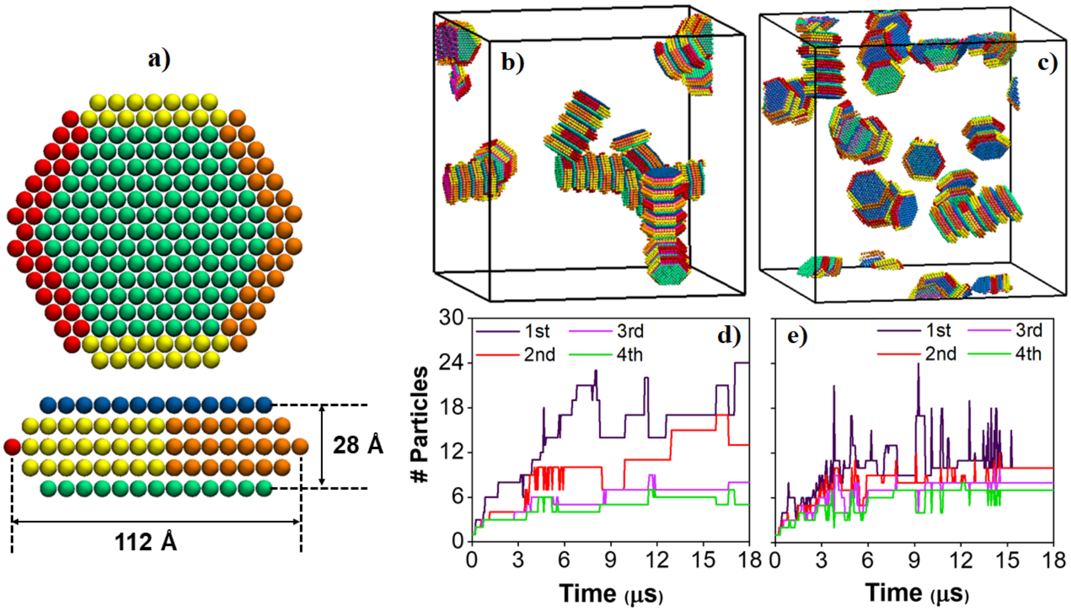

Figure S8. Schematic coarse-grained representation of large CG kaolinite nano-particle (a). Snapshots of aggregates formed in pure water (panel b) and in 1.2 M NaCl brine (c). Time evolution of the four largest clusters from simulations conducted in pure water (d) and in 1.2 M NaCl brine (e).
